# Supplementary material for: Human-interpretable image features derived from densely mapped cancer pathology slides predict diverse molecular phenotypes
Source: Nat Commun. 2021 Mar 12;12:1613. doi: 10.1038/s41467-021-21896-9 (PMC7955068; doi:10.1038/s41467-021-21896-9)
Supplement: Supplementary file 1 — Description of Additional Supplementary Files [file 41467_2021_21896_MOESM1_ESM.pdf]

Title: Supplementary Data 1.

Description: Human-interpretable image feature (HIF) clusters
