# Supplementary material for: Human-interpretable image features derived from densely mapped cancer pathology slides predict diverse molecular phenotypes
Source: Nat Commun. 2021 Mar 12;12:1613. doi: 10.1038/s41467-021-21896-9 (PMC7955068; doi:10.1038/s41467-021-21896-9)
Supplement: Supplementary file 2 — Reporting Summary [file 41467_2021_21896_MOESM2_ESM.pdf]

nature research

Corresponding author(s): Amaro Taylor-Weiner, Andrew H. Beck

Last updated by author(s): Feb 10, 2021

Reporting Summary

Nature Research wishes to improve the reproducibility of the work that we publish. This form provides structure for consistency and transparency in reporting. For further information on Nature Research policies, see our Editorial Policies and the Editorial Policy Checklist.

Statistics

For all statistical analyses, confirm that the following items are present in the figure legend, table legend, main text, or Methods section.

n/a	Confirmed	
☐	☒	The exact sample size (n) for each experimental group/condition, given as a discrete number and unit of measurement
☐	☒	A statement on whether measurements were taken from distinct samples or whether the same sample was measured repeatedly
☐	☒	The statistical test(s) used AND whether they are one- or two-sided<br>*Only common tests should be described solely by name; describe more complex techniques in the Methods section.*
☐	☒	A description of all covariates tested
☐	☒	A description of any assumptions or corrections, such as tests of normality and adjustment for multiple comparisons
☐	☒	A full description of the statistical parameters including central tendency (e.g. means) or other basic estimates (e.g. regression coefficient) AND variation (e.g. standard deviation) or associated estimates of uncertainty (e.g. confidence intervals)
☐	☒	For null hypothesis testing, the test statistic (e.g. F, t, r) with confidence intervals, effect sizes, degrees of freedom and P value noted<br>*Give P values as exact values whenever suitable.*
☒	☐	For Bayesian analysis, information on the choice of priors and Markov chain Monte Carlo settings
☒	☐	For hierarchical and complex designs, identification of the appropriate level for tests and full reporting of outcomes
☐	☒	Estimates of effect sizes (e.g. Cohen's d, Pearson's r), indicating how they were calculated

Our web collection on statistics for biologists contains articles on many of the points above.

Software and code

Policy information about availability of computer code

Data collection

TCGA histopathology images were downloaded from the publicly available database: https://www.cancer.gov/about-nci/organization/ccg/research/structural-genomics/tcga

The Cancer Imaging Archive histopathology images used for external validation can be downloaded from: https://doi.org/10.7937/TCIA.2019.4YIBTJNO

Computed human-interpretable image features (HIFs) for the five TCGA cancer types examined in this study can be downloaded from: https://github.com/Path-AI/hif2gene/tree/master/data/hifs

Data analysis

Data analyses in this study used Python and R programming languages. Source code to generate figures can be downloaded from: https://github.com/Path-AI/hif2gene. The repository also delineates the set of Python and R libraries utilized.

For manuscripts utilizing custom algorithms or software that are central to the research but not yet described in published literature, software must be made available to editors and reviewers. We strongly encourage code deposition in a community repository (e.g. GitHub). See the Nature Research guidelines for submitting code & software for further information.

Data

Policy information about availability of data

All manuscripts must include a data availability statement. This statement should provide the following information, where applicable:

- Accession codes, unique identifiers, or web links for publicly available datasets
- A list of figures that have associated raw data
- A description of any restrictions on data availability

Histopathology images were downloaded from the open TCGA database: https://www.cancer.gov/about-nci/organization/ccg/research/structural-genomics/tcga

The Cancer Imaging Archive histopathology images used for external validation can be downloaded from: https://doi.org/10.7937/TCIA.2019.4YIBTJNO

RNASeq quantifications for PD-1, PD-L1, and CTLA-4 as well as immune marker quantifications for leukocyte infiltration, TGF-Beta, IgG, and wound healing signature were obtained from the PanImmune dataset: https://gdc.cancer.gov/about-data/publications/panimmune

RNASeq quantifications for TIGIT were obtained from the PanCanAtlas dataset: https://gdc.cancer.gov/about-data/publications/pancanatlas

HRD scores were obtained from the dataset shared by Knijnenburg et al: https://gdc.cancer.gov/about-data/publications/PanCan-DDR-2018

Field-specific reporting

Please select the one below that is the best fit for your research. If you are not sure, read the appropriate sections before making your selection.

- [x] Life sciences
- [ ] Behavioural & social sciences
- [ ] Ecological, evolutionary & environmental sciences

For a reference copy of the document with all sections, see nature.com/documents/nr-reporting-summary-flat.pdf

Life sciences study design

All studies must disclose on these points even when the disclosure is negative.

Sample size	No statistical methods were used to predetermine sample size. Sample size was determined by the number of cases available in the databases used.
Data exclusions	Histopathology images were excluded if they failed basic quality control checks as determined by board-certified pathologists.
Replication	HIF-based models were validated on hold-out cohorts comprised of patients from tissue source sites not seen in the training or validation datasets.
Randomization	After the hold-out cohort was removed, nested cross-validation was used to train and validate the predictive models. Samples were randomly assigned to each of the three outer folds and five inner folds used in nested cross-validation.
Blinding	Investigators were not blinded, as no animal or human research subjects were used.

Reporting for specific materials, systems and methods

We require information from authors about some types of materials, experimental systems and methods used in many studies. Here, indicate whether each material, system or method listed is relevant to your study. If you are not sure if a list item applies to your research, read the appropriate section before selecting a response.

Materials & experimental systems

n/a	Involved in the study	
x		Antibodies
x		Eukaryotic cell lines
x		Palaeontology and archaeology
x		Animals and other organisms
x		Human research participants
x		Clinical data
x		Dual use research of concern

Methods

n/a	Involved in the study	
x		ChIP-seq
x		Flow cytometry
x		MRI-based neuroimaging
