# Supplementary material for: Human-interpretable image features derived from densely mapped cancer pathology slides predict diverse molecular phenotypes
Source: Nat Commun. 2021 Mar 12;12:1613. doi: 10.1038/s41467-021-21896-9 (PMC7955068; doi:10.1038/s41467-021-21896-9)
Supplement: Supplementary file 3 — Supplementary Information [file 41467_2021_21896_MOESM3_ESM.pdf]

Supplementary Information:

Dense, high-resolution mapping of cells and tissues from pathology images for the interpretable prediction of molecular phenotypes in cancer

Supplemental Table 1. Patient demographics and characteristics

Whole-slide images (WSIs) and clinical data were acquired from The Cancer Genome Atlas (TCGA) and encompassed BRCA, LUAD, LUSC, SKCM, and STAD patients (n=2634 total patients) from 95 distinct clinical sites. Patients with multiple samples were represented by the sample with the largest tumor region.

	**SKCM**		**STAD**		**BRCA**		**LUSC**		**LUAD**	
**Number of Patients**	327		407		1044		412		444	
**Number of Distinct Tissue Source Sites**	24		22		39		34		32	
**Age at Diagnosis (Mean +/- SD)**	57.4 +/- 15.8		64.8 +/- 10.6		58.5 +/- 13.2		67.7 +/- 8.6		65.1 +/- 10.1	
**Gender (%)**										
Male	200	61.16%	269	66.09%	12	1.15%	306	74.27%	205	46.17%
Female	127	38.84%	138	33.91%	1032	98.85%	106	25.73%	239	53.83%
**Stage (%)**										
0	4	1.22%	0	0.00%	0	0.00%	0	0.00%	0	0.00%
1	55	16.82%	92	22.60%	188	18.01%	214	51.94%	265	59.68%
2	83	25.38%	122	29.98%	595	56.99%	126	30.58%	107	24.10%
3	129	39.45%	183	44.96%	238	22.80%	68	16.50%	65	14.64%
4	14	4.28%	0	0.00%	0	0.00%	0	0.00%	0	0.00%
1-2 Not Otherwise Specified	12	3.67%	0	0.00%	0	0.00%	0	0.00%	0	0.00%
Unknown or Discrepancy	30	9.17%	10	2.46%	23	2.20%	4	0.97%	7	1.58%

Supplemental Table 2. Cell- and tissue-type annotation counts
Annotations for six cell classes and four tissue classes were obtained from a multi-hundred pathologist network. Cell type annotations are individual pixels while tissue region annotations are fully connected components of pixels. The total number of cell and tissue type annotations collected on TCGA H&E images and several additional H&E datasets in aggregate are shown broken down by cancer type and annotation type. In total, 5,719 WSIs curated from TCGA and additional datasets were used during development of the deep learning models (training and validation). Tissue-level segmentations and cell-level predictions were then generated on 2,826 TCGA WSIs.

**TCGA Datasets**						
**Cell-Level Annotations**	BRCA	LUAD	LUSC	SKCM	STAD	Total
Cancer Cell	25561	5077	24117	43200	18949	116904
Fibroblast	7464	3367	8833	10994	9155	39813
Lymphocyte	7904	4223	9211	17247	11388	49973
Macrophage	3062	3474	7758	5596	4297	24187
Plasma Cell	1792	2689	6997	5990	5183	22651
Other Cell / Background	12057	3128	11632	22069	20626	69512
Total	57840	21958	68548	105096	69598	**323040**
**Tissue-Level Annotations**						
Cancer Tissue	3660	2549	5433	7797	3800	23239
Cancer-Associated Stroma	2001	1696	4591	2287	2170	12745
Necrosis	152	318	1335	991	431	3227
Normal / Background	1824	992	2467	8536	2516	16335
Total	7637	5555	13826	19611	8917	**55546**
**Whole-Slide Images**						
Development	359	137	307	358	400	1561
Inference	1117	501	443	358	407	2826
QC Excluded	20	37	24	5	5	91
Total	1137	538	467	363	412	**2917**
**Additional Datasets**						
**Cell-Level Annotations**	BRCA	LUAD	LUSC	SKCM	STAD	Total
Cancer Cell	40518	101979	0	254392	9435	406324
Fibroblast	9201	38603	2773	49815	3106	103498
Lymphocyte	23145	65223	21852	125842	3816	239878
Macrophage	5422	17424	9723	49879	987	83435

Plasma Cell	6797	6401	5487	19158	1091	38934
Other Cell / Background	45391	28336	5226	159118	9564	247635
Total	130474	257966	45061	658204	27999	**1119704**
**Tissue-Level Annotations**						
Cancer Tissue	9945	14654	6471	27889	4428	63387
Cancer-Associated Stroma	6617	8826	3866	14076	2257	35642
Necrosis	741	1279	1256	2843	326	6445
Normal / Background	5791	14436	2752	16446	1119	40544
Total	23094	39195	14345	61254	8130	**146018**
**Whole-Slide Images**						
Development	698	1908	438	1002	112	4158
Inference	0	0	0	0	0	0
Total	698	1908	438	1002	112	**4158**
**Combined**						
	BRCA	LUAD	LUSC	SKCM	STAD	Total
**Total Cell-Level Annotations**	188314	279924	113609	763300	97597	**1442744**
**Total Tissue-Level Annotations**	30731	44750	28171	80865	17047	**201564**
**Total Whole-Slide Images**	1835	2446	905	1365	524	**7075**

Supplemental Table 3. Validation of human-interpretable image features (HIFs) against immune markers broken down by cancer type

The top three HIF clusters, ranked by median absolute Spearman correlation ρ (computed across HIFs in a given cluster), for each of four immune markers (leukocyte infiltration, IgG expression, TGF-β expression, wound healing signature), angiogenesis, signature, hypoxia signature, and a negative control (Case ID). Interquartile ranges (IQR) are also shown. Q-values are combined (via the Empirical Brown's method) and corrected (via Benjamini-Hochberg procedure) P-values per cluster. In correlation analyses of immune markers, angiogenesis signature, and the negative control, SKCM-specific analyses were excluded due to insufficient sample sizes (although SKCM was included in pan-cancer correlations). In correlation analysis of hypoxia signature, STAD was excluded since hypoxia data on the cancer type was unavailable. Clusters were defined pan-cancer to enable comparisons across cancer types. Notably, we can observe high concordance among the top-correlated HIF clusters per immune marker across cancer types. HIF clusters are provided in Supplemental Data 1.

**Leukocyte Infiltration Signature**			
Cancer Type	HIF Cluster	Median (IQR) Abs(ρ)	Q-Value
BRCA (n=1022)	10	0.48 (0.45, 0.51)	1.37E-125
	9	0.41 (0.36, 0.47)	3.85E-166
	7	0.37 (0.33, 0.43)	8.97E-141
LUAD (n=390)	10	0.49 (0.41, 0.51)	5.00E-23
	9	0.39 (0.29, 0.43)	1.07E-12
	7	0.27 (0.17, 0.33)	1.86E-29
LUSC (n=400)	9	0.40 (0.36, 0.44)	6.79E-13
	10	0.38 (0.33, 0.43)	4.06E-12
	4	0.22 (0.20, 0.27)	3.51E-17
STAD (n=328)	10	0.36 (0.32, 0.43)	1.27E-11
	8	0.35 (0.32, 0.38)	6.42E-08
	12	0.27 (0.23, 0.33)	1.31E-07
Pan-Cancer (n=2202)	10	0.48 (0.45, 0.53)	8.48E-279
	9	0.46 (0.43, 0.49)	2.09E-280
	7	0.40 (0.33, 0.42)	1.09E-295

IgG Expression

Cancer Type	HIF Cluster	Median (IQR) Abs(ρ)	Q-Value
BRCA (n=1022)	9	0.44 (0.40, 0.49)	3.05E-194
	10	0.44 (0.41, 0.46)	6.62E-100
	8	0.37 (0.20, 0.41)	3.58E-171
LUAD (n=390)	9	0.34 (0.23, 0.40)	1.30E-09
	10	0.25 (0.22, 0.27)	2.14E-08
	4	0.17 (0.13, 0.20)	7.04E-10
LUSC (n=400)	9	0.36 (0.29, 0.44)	1.03E-15
	10	0.26 (0.18, 0.31)	5.09E-09
	4	0.24 (0.18, 0.26)	1.03E-15
STAD (n=328)	8	0.35 (0.32, 0.40)	2.39E-09
	9	0.32 (0.24, 0.37)	1.14E-06
	10	0.26 (0.23, 0.29)	7.62E-05
Pan-Cancer (n=2202)	9	0.58 (0.53, 0.60)	2.67E-204
	7	0.44 (0.38, 0.49)	4.83E-54
	10	0.44 (0.38, 0.47)	4.86E-42

TGF-β Signature

Cancer Type	HIF Cluster	Median (IQR) Abs(ρ)	Q-Value
BRCA (n=1022)	12	0.37 (0.24, 0.38)	7.30E-85
	14	0.35 (0.27, 0.43)	3.90E-75
	4	0.32 (0.22, 0.35)	1.55E-195
LUAD (n=390)	4	0.26 (0.22, 0.28)	5.57E-10
	14	0.23 (0.10, 0.25)	1.38E-06
	12	0.20 (0.16, 0.23)	3.85E-06
LUSC (n=400)	14	0.30 (0.26, 0.36)	1.81E-10
	12	0.27 (0.23, 0.30)	4.66E-13
	4	0.25 (0.16, 0.26)	2.04E-08

STAD (n=328)	12	0.40 (0.36, 0.41)	7.00E-16
	14	0.33 (0.26, 0.38)	3.26E-06
	4	0.31 (0.21, 0.36)	5.60E-12
Pan-Cancer (n=2202)	4	0.31 (0.21, 0.32)	1.03E-90
	14	0.28 (0.24, 0.31)	4.09E-104
	12	0.25 (0.22, 0.29)	7.62E-104

**Wound Healing Signature**			
Cancer Type	HIF Cluster	Median (IQR) Abs(ρ)	Q-Value
BRCA (n=1022)	16	0.37 (0.36, 0.42)	9.84E-76
	2	0.25 (0.29, 0.41)	3.42E-74
	9	0.29 (0.16, 0.35)	3.42E-74
LUAD (n=390)	16	0.15 (0.14, 0.18)	0.19
	2	0.13 (0.08, 0.19)	9.39E-08
	20	0.10 (0.07, 0.14)	1.84E-02
LUSC (n=400)	2	0.19 (0.14, 0.22)	1.56E-04
	12	0.11 (0.10, 0.15)	1.87E-03
	1	0.09 (0.07, 0.11)	1.82E-02
STAD (n=328)	12	0.42 (0.41, 0.47)	1.73E-18
	4	0.40 (0.24, 0.47)	4.76E-20
	14	0.32 (0.28, 0.38)	6.29E-08
Pan-Cancer (n=2202)	16	0.35 (0.27, 0.38)	1.80E-167
	2	0.34 (0.25, 0.40)	3.85E-141
	12	0.29 (0.24, 0.36)	7.59E-96

**Angiogenesis Signature**			
Cancer Type	HIF Cluster	Median (IQR) Abs(ρ)	Q-Value
BRCA (n=1022)	12	0.336 (0.303, 0.362)	1.86E-92
	16	0.322 (0.294, 0.387)	1.88E-284
	4	0.273 (0.106, 0.361)	1.44E-168
LUAD	12	0.154 (0.089, 0.174)	7.69E-02

(n=390)	4	0.133 (0.118, 0.176)	1.22E-2
	2	0.109 (0.073, 0.162)	2.99E-12
LUSC (n=400)	4	0.183 (0.126, 0.207)	3.97E-4
	12	0.148 (0.106, 0.172)	1.07E-2
	7	0.146 (0.110, 0.175)	3.97E-4
STAD (n=328)	12	0.450 (0.404, 0.494)	6.42E-22
	4	0.421 (0.260, 0.503)	1.15E-21
	14	0.339 (0.287, 0.419)	3.64E-15
Pan-Cancer (n=2202)	12	0.316 (0.288, 0.373)	1.46E-186
	16	0.313 (0.240, 0.368)	1.14E-126
	2	0.308 (0.252, 0.321)	1.59E-63

**Hypoxia Score**			
Cancer Type	HIF Cluster	Median (IQR) Abs(ρ)	Q-Value
BRCA (n=1033)	16	0.361 (0.352, 0.405)	5.71E-79
	2	0.340 (0.286, 0.416)	1.41E-58
	9	0.249 (0.121, 0.328)	2.54E-29
LUAD (n=437)	2	0.152 (0.08, 0.241)	7.93E-8
	10	0.121 (0.06, 0.130)	6.43E-3
	19	0.09 (0.06, 0.105)	8.29E-12
LUSC (n=410)	2	0.158 (0.124, 0.243)	2.42E-5
	14	0.120 (0.08, 0.143)	4.50E-3
	18	0.107 (0.08, 0.112)	2.29E-1
SKCM (n=316)	2	0.209 (0.150, 0.236)	2.71E-5
	15	0.157 (0.120, 0.157)	5.12E-6
	10	0.152 (0.120, 0.189)	2.42E-4
Pan-Cancer (n=2196)	2	0.452 (0.331, 0.487)	1.84E-292
	9	0.364 (0.270, 0.398)	6.11E-235
	16	0.355 (0.282, 0.425)	3.83E-72

**Case ID (Negative Control)**

Cancer Type	HIF Cluster	Median (IQR) Abs(ρ)	Q-Value
BRCA (n=1022)	17	0.07 (0.06, 0.07)	0.36
	19	0.05 (0.04, 0.06)	0.68
	13	0.04 (0.02, 0.04)	0.86
LUAD (n=390)	19	0.17 (0.10, 0.21)	0.20
	7	0.06 (0.03, 0.08)	0.78
	14	0.05 (0.03, 0.07)	0.78
LUSC (n=400)	13	0.10 (0.01, 0.12)	0.62
	1	0.09 (0.07, 0.11)	0.26
	2	0.08 (0.07, 0.14)	0.88
STAD (n=328)	18	0.07 (0.04, 0.07)	0.92
	1	0.07 (0.04, 0.09	0.99
	12	0.06 (0.03, 0.08)	0.92
Pan-Cancer (n=2202)	17	0.06 (0.04, 0.06)	0.08
	19	0.05 (0.03, 0.05)	0.08
	5	0.02 (0.01, 0.04)	0.40

Supplemental Table 4. Holdout set definition

Hold-out sets were defined as approximately 20-30% of all samples and consisted of 2-3 tissue source sites not included in the training set. This enabled us to evaluate our final ensemble models on varying patient demographics and institutions. The pan-cancer hold-out set was defined as the concatenation of hold-out sets from individual cancer types. The percentages of positive labels in the training and hold-out sets are shown. For a given cancer type, the same set of hold-out tissue source sites was used across prediction outcomes.

<table>
<tr><th>Cancer Type</th><th>Tissue Source Site / Code</th><th>Hold-Out N</th><th>Immune Score</th><th>% Positive Train</th><th>% Positive Hold-Out</th></tr>
<tr><td rowspan="5">BRCA</td><td rowspan="5">BH (University of Pittsburgh)
A2 (Walter Reed)</td><td rowspan="3">239/1022
(23.4%)</td><td>PD-1</td><td>45.8%</td><td>48.1%</td></tr>
<tr><td>PDL-1</td><td>47.9%</td><td>51.0%</td></tr>
<tr><td>CTLA-4</td><td>53.3%</td><td>51.5%</td></tr>
<tr><td>208/904
(23.0%)</td><td>HRD</td><td>16.7%</td><td>12.5%</td></tr>
<tr><td>201/977
(20.6%)</td><td>TIGIT</td><td>48.8%</td><td>49.3%</td></tr>
<tr><td rowspan="5">LUAD</td><td rowspan="5">55 (International Genomics Consortium)
50 (University of Pittsburgh)</td><td rowspan="3">95/390
(24.4%)</td><td>PD-1</td><td>46.1%</td><td>51.6%</td></tr>
<tr><td>PDL-1</td><td>57.6%</td><td>63.2%</td></tr>
<tr><td>CTLA-4</td><td>51.9%</td><td>53.7%</td></tr>
<tr><td>104/417
(24.9%)</td><td>HRD</td><td>26.2%</td><td>19.2%</td></tr>
<tr><td>100/420
(23.8%)</td><td>TIGIT</td><td>50.3%</td><td>60.0%</td></tr>
<tr><td rowspan="5">LUSC</td><td rowspan="5">85 (Asterand)
66 (Indivumed)
33 (Johns Hopkins)</td><td rowspan="3">98/400
(24.5%)</td><td>PD-1</td><td>48.7%</td><td>42.9%</td></tr>
<tr><td>PDL-1</td><td>35.8%</td><td>36.7%</td></tr>
<tr><td>CTLA-4</td><td>34.7%</td><td>37.7%</td></tr>
<tr><td>96/384
(25.0%)</td><td>HRD</td><td>18.8%</td><td>19.8%</td></tr>
<tr><td>98/398
(24.6%)</td><td>TIGIT</td><td>33.0%</td><td>30.6%</td></tr>
<tr><td>SKCM</td><td>D3 (MD Anderson)
WE (Norfolk and Norwich Hospital)</td><td>83/316
(26.3%)</td><td>TIGIT</td><td>57.5%</td><td>63.9%</td></tr>
<tr><td rowspan="5">STAD</td><td rowspan="5">VQ (Barretos Cancer Hospital)
RD (Peter MacCallum Cancer Center)
FP (International Genomics Consortium)</td><td rowspan="3">87/328
(26.5%)</td><td>PD-1</td><td>52.7%</td><td>49.4%</td></tr>
<tr><td>PDL-1</td><td>42.3%</td><td>42.5%</td></tr>
<tr><td>CTLA-4</td><td>61.4%</td><td>65.5%</td></tr>
<tr><td>80/316
(25.3%)</td><td>HRD</td><td>28.0%</td><td>50.0%</td></tr>
<tr><td>87/341
(25.5%)</td><td>TIGIT</td><td>66.9%</td><td>63.2%</td></tr>
<tr><td rowspan="2">Pan-Cancer</td><td rowspan="2">Concatenated
(BRCA, LUAD, LUSC, STAD)</td><td rowspan="2">519/2140
(24.3%)</td><td>PD-1</td><td>47.4%</td><td>48.0%</td></tr>
<tr><td>PDL-1</td><td>46.6%</td><td>49.1%</td></tr>
</table>

			CTLA-4	51.3%	51.1%
		488/2021 (24.1%)	HRD	20.7%	21.5%
	Concatenated (BRCA, LUAD, LUSC, SKCM, STAD)	569/2452 (23.2%)	TIGIT	50.1%	52.2%

Supplemental Table 5. Full cross-validation and hold-out results from predicting clinically-relevant phenotypes using HIFs

Supervised prediction was conducted on binarized high versus low expression of five clinically-relevant molecular phenotypes: (1) PD-1 expression, (2) PD-L1 expression, (3) CTLA-4 expression, (4) HRD score, and (5) TIGIT expression. SKCM predictions were conducted only for TIGIT expression due to insufficient labels for the remaining phenotypes. Pan-cancer analyses used the same binary labels thresholded independently by cancer type. Pan-cancer predictions for TIGIT included all five cancer types, while pan-cancer predictions for the remaining phenotypes included the four cancer types excluding SKCM. Cross-validation (CV) and hold-out performance were measured by area under the receiver operating characteristic (AUROC) and area under the precision-recall curve (AUPRC). Null classifiers correspond to AUROC=0.50 and AUPRC=% positive labels. CV metrics are presented as the mean and standard deviation computed across three models, each trained on two of the three outer folds and evaluated on the third in accordance with nested CV. 95% confidence intervals and p-values for hold-out metrics were computed with the DeLong method and with the empirical bootstrap method (n=1000). P-values were adjusted using the Benjamini-Hochberg procedure, denoted as q-values below. Immune checkpoint protein, HRD, and TIGIT labels were curated from distinct datasets, resulting in differing sample sizes for the different tasks.

		Cross Validation Metrics			Hold-Out Metrics			
Cancer Type (Training, Hold-Out)	Immune Score	Mean AUROC	Mean AUPRC	% Positive	AUROC (95% CI, DeLong)	AUROC (95% CI, bootstrap)	AUPRC (95% CI, bootstrap)	% Positive
BRCA n=783, 239	PD-1	0.773 +/- 0.01	0.757 +/- 0.01	45.8%	0.777 (0.718, 0.836) $q=2.49x10^{-19}$	0.777 (0.716, 0.835) q<0.002	0.778 (0.696, 0.851) q<0.002	48.1%
	PD-L1	0.729 +/- 0.02	0.714 +/- 0.01	47.9%	0.663 (0.594, 0.731) $q=6.65x10^{-6}$	0.663 (0.594, 0.733) q<0.002	0.702 (0.618, 0.775) q<0.002	51.0%
	CTLA-4	0.842 +/- 3E-3	0.860 +/- 5E-3	53.3%	0.794 (0.737, 0.851) $q=3.65x10^{-23}$	0.794 (0.739, 0.848) q<0.002	0.818 (0.752, 0.875) q<0.002	51.5%
n=696, 208	HRD	0.743 +/- 0.01	0.393 +/- 0.02	16.7%	0.773 (0.669, 0.878) $q=6.95x10^{-7}$	0.773 (0.665, 0.867) q<0.002	0.354 (0.196, 0.570) q<0.002	12.5%
n=776, 201	TIGIT	0.793 +/- 0.02	0.804 +/- 0.01	48.8%	0.788 (0.725, 0.850) $q=1.12x10^{-18}$	0.788 (0.723, 0.848) q<0.002	0.799 (0.724, 0.865) q<0.002	49.3%
LUAD n=295, 95	PD-1	0.814 +/- 0.03	0.786 +/- 0.02	46.1%	0.712 (0.607, 0.817) $q=1.25x10^{-4}$	0.712 (0.593, 0.805) q<0.002	0.726 (0.587, 0.849) q=0.004	51.6%

	PD-L1	0.811 +/- 0.03	0.849 +/- 0.02	57.6%	0.726 (0.618, 0.834) q=8.20x10^{-5}	0.726 (0.610, 0.832) q<0.002	0.776 (0.653, 0.903) q=0.019	63.2%
	CTLA-4	0.781 +/- 0.02	0.802 +/- 0.01	51.9%	0.718 (0.615, 0.820) q=6.21x10^{-5}	0.718 (0.605, 0.808) q<0.002	0.756 (0.634, 0.849) q<0.002	53.7%
n=313 ,104	HRD	0.594 +/- 0.04	0.325 +/- 0.06	26.2%	0.552 (0.417, 0.688) q=0.484	0.552 (0.406, 0.685) q=0.257	0.209 (0.119, 0.375) q=0.339	19.2%
n= 320, 100	TIGIT	0.732 +/- 0.03	0.734 +/- 0.05	50.3%	0.766 (0.674, 0.858) q=5.28x10^{-8}	0.766 (0.670, 0.852) q<0.002	0.848 (0.766, 0.911) q<0.002	60.0%
LUSC n=302, 98	PD-1	0.766 +/- 0.04	0.773 +/- 0.04	48.7%	0.680 (0.574, 0.787) q=1.34x10^{-3}	0.680 (0.571, 0.775) q=0.003	0.611 (0.459, 0.745) q=0.019	42.9%
	PD-L1	0.650 +/- 0.02	0.518 +/- 0.02	35.8%	0.521 (0.396, 0.646) q=0.773	0.521 (0.394, 0.637) q=0.436	0.425 (0.292, 0.575) q=0.222	36.7%
	CTLA-4	0.760 +/- 0.01	0.663 +/- 0.01	34.7%	0.694 (0.577, 0.811) q=1.57x10^{-3}	0.694 (0.571, 0.798) q<0.002	0.541 (0.377, 0.706) q=0.033	37.7%
n=288, 96	HRD	0.564 +/- 0.02	0.228 +/- 0.05	18.8%	0.482 (0.345, 0.619) q=0.797	0.482 (0.338, 0.606) q=0.646	0.178 (0.106, 0.295) q=0.658	19.8%
n=300, 98	TIGIT	0.703 +/- 0.01	0.559 +/- 0.03	33.0%	0.703 (0.591, 0.815) q=6.12x10^{-4}	0.703 (0.597, 0.811) q<0.002	0.494 (0.329, 0.679) q=0.018	30.6%
SKCM n=233, 83	TIGIT	0.854 +/- 0.05	0.895 +/- 0.03	57.5%	0.860 (0.762, 0.958) q=2.62x10^{-12}	0.860 (0.771, 0.954) q<0.002	0.871 (0.757, 0.976) q<0.002	63.9%
STAD n=241, 87	PD-1	0.826 +/- 0.02	0.847 +/- 0.01	52.7%	0.684 (0.581, 0.803) q=1.11x10^{-3}	0.684 (0.566, 0.790) q=0.003	0.701 (0.541, 0.825) q=0.007	49.4%
	PD-L1	0.838 +/- 0.06	0.794 +/- 0.06	42.3%	0.596 (0.462, 0.729) q=0.182	0.596 (0.470, 0.733) q=0.068	0.616 (0.457, 0.767) q=0.016	42.5%
	CTLA-4	0.799 +/- 0.03	0.856 +/- 0.01	61.4%	0.626 (0.507, 0.758) q=0.048	0.626 (0.498, 0.746) q=0.033	0.759 (0.638, 0.867) q=0.046	65.5%
n=236, 80	HRD	0.681 +/- 0.03	0.433 +/- 0.07	28.0%	0.628 (0.504, 0.754) q=0.051	0.628 (0.501, 0.750) q=0.031	0.647 (0.494, 0.797) q=0.038	50.0%
n=254, 87	TIGIT	0.674 +/- 0.03	0.764 +/- 0.03	66.9%	0.657 (0.528, 0.771) q=0.021	0.657 (0.536, 0.779) q=0.004	0.756 (0.617, 0.882) q=0.045	63.2%
Pan-Cancer n=1621, 519	PD-1	0.718 +/- 0.03	0.691 +/- 0.04	47.4%	0.677 (0.631, 0.722) q=1.93x10^{-13}	0.677 (0.636, 0.723) q<0.002	0.673 (0.615, 0.731) q<0.002	48.0%

	PD-L1	0.670 +/- 0.04	0.643 +/- 0.04	46.6%	0.626 (0.577, 0.674) q=7.56x10^{-7}	0.626 (0.574, 0.674) q<0.002	0.639 (0.574, 0.707) q<0.002	49.1%
	CTLA-4	0.727 +/- 0.05	0.732 +/- 0.06	51.3%	0.738 (0.695, 0.780) q=1.67x10^{-26}	0.738 (0.697, 0.785) q<0.002	0.752 (0.699, 0.807) q<0.002	51.1%
n=1533, 488	HRD	0.640 +/- 0.02	0.293 +/- 0.02	20.7%	0.651 (0.593, 0,707) q=6.12x10^{-7}	0.651 (0.596, 0,709) q<0.002	0.336 (0.263, 0.421) q<0.002	21.5%
n=1883, 569	TIGIT	0.771 +/- 0.01	0.770 +/- 0.01	50.1%	0.792 (0.754, 0.828) q=1.21x10^{-52}	0.792 (0.754, 0.827) q<0.002	0.799 (0.750, 0.844) q<0.002	52.2%

Supplemental Table 6. End-to-end (E2E) model benchmarks

Comparison of AUROC hold-out metrics between HIF-based linear models (results reproduced from Supplemental Table 5) and conventional end-to-end models. We trained 26 distinct CNNs for each of the 26 molecular phenotype prediction tasks described above using single-instance learning, using the computationally-efficient ShuffleNet architecture. The same training and hold-out sets were used in development of both types of models.

		Hold-Out Metrics		
		E2E Mode	HIF-Based	
Cancer Type (Training, Hold-Out)	Immune Score	AUROC	AUROC (95% CI, DeLong)	AUROC (95% CI, bootstrap)
BRCA<br>n=783, 239	PD-1	0.71	0.777<br>(0.718, 0.836)<br>$q=2.49x10^{-19}$	0.777<br>(0.716, 0.835)<br>q<0.002
	PD-L1	0.62	0.663<br>(0.594, 0.731)<br>$q=6.65x10^{-6}$	0.663<br>(0.594, 0.733)<br>q<0.002
	CTLA-4	0.71	0.794<br>(0.737, 0.851)<br>$q=3.65x10^{-23}$	0.794<br>(0.739, 0.848)<br>q<0.002
n=696, 208	HRD	0.80	0.773<br>(0.669, 0.878)<br>$q=6.95x10^{-7}$	0.773<br>(0.665, 0.867)<br>q<0.002
n=776, 201	TIGIT	0.73	0.788<br>(0.725, 0.850)<br>$q=1.12x10^{-18}$	0.788<br>(0.723, 0.848)<br>q<0.002
LUAD<br>n=295, 95	PD-1	0.67	0.712<br>(0.607, 0.817)<br>$q=1.25x10^{-4}$	0.712<br>(0.593, 0.805)<br>q<0.002
	PD-L1	0.73	0.726<br>(0.618, 0.834)<br>$q=8.20x10^{-5}$	0.726<br>(0.610, 0.832)<br>q<0.002
	CTLA-4	0.71	0.718<br>(0.615, 0.820)<br>$q=6.21x10^{-5}$	0.718<br>(0.605, 0.808)<br>q<0.002
n=313 ,104	HRD	0.71	0.552<br>(0.417, 0.688)<br>q=0.484	0.552<br>(0.406, 0.685)<br>q=0.257
n= 320, 100	TIGIT	0.70	0.766<br>(0.674, 0.858)<br>$q=5.28x10^{-8}$	0.766<br>(0.670, 0.852)<br>q<0.002
LUSC	PD-1	0.69	0.680	0.680

n=302, 98			(0.574, 0.787) q=1.34×10^{-3}	(0.571, 0.775) q=0.003
	PD-L1	0.59	0.521 (0.396, 0.646) q=0.773	0.521 (0.394, 0.637) q=0.436
	CTLA-4	0.56	0.694 (0.577, 0.811) q=1.57×10^{-3}	0.694 (0.571, 0.798) q<0.002
n=288, 96	HRD	0.63	0.482 (0.345, 0.619) q=0.797	0.482 (0.338, 0.606) q=0.646
n=300, 98	TIGIT	0.63	0.703 (0.591, 0.815) q=6.12×10^{-4}	0.703 (0.597, 0.811) q<0.002
SKCM n=233, 83	TIGIT	0.61	0.860 (0.762, 0.958) q=2.62×10^{-12}	0.860 (0.771, 0.954) q<0.002
STAD n=241, 87	PD-1	0.62	0.684 (0.581, 0.803) q=1.11×10^{-3}	0.684 (0.566, 0.790) q=0.003
	PD-L1	0.62	0.596 (0.462, 0.729) q=0.182	0.596 (0.470, 0.733) q=0.068
	CTLA-4	0.65	0.626 (0.507, 0.758) q=0.048	0.626 (0.498, 0.746) q=0.033
n=236, 80	HRD	0.56	0.628 (0.504, 0.754) q=0.051	0.628 (0.501, 0.750) q=0.031
n=254, 87	TIGIT	0.59	0.657 (0.528, 0.771) q=0.021	0.657 (0.536, 0.779) q=0.004
Pan-Cancer n=1621, 519	PD-1	0.66	0.677 (0.631, 0.722) q=1.93×10^{-13}	0.677 (0.636, 0.723) q<0.002
	PD-L1	0.61	0.626 (0.577, 0.674) q=7.56×10^{-7}	0.626 (0.574, 0.674) q<0.002
	CTLA-4	0.67	0.738 (0.695, 0.780) q=1.67×10^{-26}	0.738 (0.697, 0.785) q<0.002
n=1533, 488	HRD	0.69	0.651 (0.593, 0,707) q=6.12×10^{-7}	0.651 (0.596, 0,709) q<0.002
n=1883, 569	TIGIT	0.67	0.792 (0.754, 0.828) q=1.21×10^{-52}	0.792 (0.754, 0.827) q<0.002

Supplemental Table 7. Random forest HIF-based model comparison

Comparison of AUROC and AUPRC performance between HIF-based linear models (results reproduced from Supplemental Table 5) and HIF-based random forest models across the 26 molecular phenotype prediction tasks. Random forest models account for interaction terms, and can thus test the hypothesis that capturing interactions between the 607 HIFs can improve model performance. The same training and hold-out sets were used in development of both types of models.

		Random Forest HIF-Based Hold-Out Metrics		Sparse Group Lasso HIF-Based Hold-Out Metrics			
Cancer Type (Training, Hold-Out)	Immune Score	AUROC	AUPRC	AUROC (95% CI, DeLong)	AUROC (95% CI, bootstrap)	AUPRC (95% CI, bootstrap)	% Positive
BRCA n=783, 239	PD-1	0.786	0.770	0.777<br>(0.718, 0.836)<br>$q=2.49\times10^{-19}$	0.777<br>(0.716, 0.835)<br>q<0.002	0.778<br>(0.696, 0.851)<br>q=0.002	48.1%
	PD-L1	0.657	0.706	0.663<br>(0.594, 0.731)<br>$q=6.65\times10^{-6}$	0.663<br>(0.594, 0.733)<br>q<0.002	0.702<br>(0.618, 0.775)<br>q=0.002	51.0%
	CTLA-4	0.779	0.806	0.794<br>(0.737, 0.851)<br>$q=3.65\times10^{-23}$	0.794<br>(0.739, 0.848)<br>q<0.002	0.818<br>(0.752, 0.875)<br>q=0.002	51.5%
n=696, 208	HRD	0.749	0.305	0.773<br>(0.669, 0.878)<br>$q=6.95\times10^{-7}$	0.773<br>(0.665, 0.867)<br>q<0.002	0.354<br>(0.196, 0.570)<br>q=0.002	12.5%
n=776, 201	TIGIT	0.812	0.817	0.788<br>(0.725, 0.850)<br>$q=1.12\times10^{-18}$	0.788<br>(0.723, 0.848)<br>q<0.002	0.799<br>(0.724, 0.865)<br>q=0.002	49.3%
LUAD n=295, 95	PD-1	0.673	0.721	0.712<br>(0.607, 0.817)<br>$q=1.25\times10^{-4}$	0.712<br>(0.593, 0.805)<br>q<0.002	0.726<br>(0.587, 0.849)<br>q=0.004	51.6%
	PD-L1	0.646	0.727	0.726<br>(0.618, 0.834)<br>$q=8.20\times10^{-5}$	0.726<br>(0.610, 0.832)<br>q<0.002	0.776<br>(0.653, 0.903)<br>q=0.019	63.2%
	CTLA-4	0.706	0.728	0.718<br>(0.615, 0.820)<br>$q=6.21\times10^{-5}$	0.718<br>(0.605, 0.808)<br>q<0.002	0.756<br>(0.634, 0.849)<br>q=0.002	53.7%
n=313 ,104	HRD	0.492	0.173	0.552<br>(0.417, 0.688)<br>q=0.484	0.552<br>(0.406, 0.685)<br>q=0.257	0.209<br>(0.119, 0.375)<br>q=0.339	19.2%
n= 320, 100	TIGIT	0.753	0.822	0.766<br>(0.674, 0.858)<br>$q=5.28\times10^{-8}$	0.766<br>(0.670, 0.852)<br>q<0.002	0.848<br>(0.766, 0.911)<br>q=0.002	60.0%
LUSC n=302, 98	PD-1	0.720	0.746	0.680<br>(0.574, 0.787)<br>$q=1.34\times10^{-3}$	0.680<br>(0.571, 0.775)<br>q=0.003	0.611<br>(0.459, 0.745)<br>q=0.019	42.9%
	PD-L1	0.612	0.510	0.521	0.521	0.425	36.7%

				(0.396, 0.646) q=0.773	(0.394, 0.637) q=0.436	(0.292, 0.575) q=0.222	
	CTLA-4	0.677	0.540	0.694 (0.577, 0.811) $q=1.57\times10^{-3}$	0.694 (0.571, 0.798) q<0.002	0.541 (0.377, 0.706) q=0.033	37.7%
n=288, 96	HRD	0.555	0.218	0.482 (0.345, 0.619) q=0.797	0.482 (0.338, 0.606) q=0.646	0.178 (0.106, 0.295) q=0.658	19.8%
n=300, 98	TIGIT	0.727	0.549	0.703 (0.591, 0.815) $q=6.12\times10^{-4}$	0.703 (0.597, 0.811) q<0.002	0.494 (0.329, 0.679) q=0.018	30.6%
SKCM n=233, 83	TIGIT	0.853	0.851	0.860 (0.762, 0.958) $q=2.62\times10^{-12}$	0.860 (0.771, 0.954) q<0.002	0.871 (0.757, 0.976) q=0.002	63.9%
STAD n=241, 87	PD-1	0.586	0.566	0.684 (0.581, 0.803) $q=1.11\times10^{-3}$	0.684 (0.566, 0.790) q=0.003	0.701 (0.541, 0.825) q=0.007	49.4%
	PD-L1	0.588	0.536	0.596 (0.462, 0.729) q=0.182	0.596 (0.470, 0.733) q=0.068	0.616 (0.457, 0.767) q=0.016	42.5%
	CTLA-4	0.597	0.710	0.626 (0.507, 0.758) q=0.048	0.626 (0.498, 0.746) q=0.033	0.759 (0.638, 0.867) q=0.046	65.5%
n=236, 80	HRD	0.624	0.663	0.628 (0.504, 0.754) q=0.051	0.628 (0.501, 0.750) q=0.031	0.647 (0.494, 0.797) q=0.038	50.0%
n=254, 87	TIGIT	0.613	0.655	0.657 (0.528, 0.771) q=0.021	0.657 (0.536, 0.779) q=0.004	0.756 (0.617, 0.882) q=0.045	63.2%

Supplemental Table 8. Combined and corrected P-values for predictive HIF clusters

One-sided P-values associated with individual HIFs were computed based on a 1000-iteration permutation test. Individual HIF P-values were combined via the Empirical Brown's Method into cluster-level P-values and corrected using the Benjamini-Hochberg procedure (see Statistical Analysis in Methods for more details). Q-values (combined and corrected cluster-level P-values) are shown broken down by cancer type and prediction task. HIF clusters with no non-zero ensemble betas are grayed. The top five predictive HIF clusters per prediction (or fewer in the case of ensemble models with <5 non-zero clusters) are bolded.

Pan-Cancer					
Cluster Number	PD-1 Prediction Q-Value	PD-L1 Prediction Q-Value	CTLA-4 Prediction Q-Value	HRD Prediction Q-Value	TIGIT Prediction Q-Value
1	**1.64E-03**	**3.51E-02**	**5.00E-02**		**5.84E-02**
2		**2.64E-04**	4.90E-05	**2.26E-04**	
3	2.90E-01	3.80E-03	2.16E-04		**1.38E-03**
4		2.63E-02	1.72E-06	**4.36E-12**	
5		**7.04E-08**	8.17E-05		
6	1.50E-02	7.62E-02	1.78E-11		
7	**2.40E-01**	**4.84E-03**	2.11E-01		
8	**1.20E-01**	7.84E-01	6.22E-03		
9	**1.40E-02**	3.74E-01	4.87E-01	**6.94E-04**	
10	**1.24E-03**	**5.14E-04**	**2.65E-04**		**6.49E-02**
11		8.36E-03	**3.80E-07**		
12		4.79E-03	2.67E-04	**3.52E-12**	
13		9.41E-08	1.58E-10	1.82E-06	
14		5.95E-04	7.22E-05		
15			**5.27E-20**		
16	5.88E-01	6.12E-01	1.15E-06	**1.88E-04**	
17					
18					
19		1.71E-02	7.17E-07		
20		4.84E-03	**3.18E-05**		

BRCA

Cluster Number	PD-1 Prediction Q-Value	PD-L1 Prediction Q-Value	CTLA-4 Prediction Q-Value	HRD Prediction Q-Value	TIGIT Prediction Q-Value
1	**9.51E-02**	**4.42E-02**	9.05E-03	1.55E-02	**3.15E-01**
2		7.49E-03	9.05E-03	**4.12E-04**	
3		1.28E-05	**4.46E-10**	1.65E-07	1.18E-03
4		1.12E-01	6.67E-08	3.08E-05	**1.34E-01**
5		6.90E-02	1.73E-02	1.65E-02	7.30E-01
6		5.10E-04		9.06E-07	
7		1.02E-08	2.24E-03	1.91E-03	**1.98E-03**
8	**9.51E-02**	**4.13E-07**	**1.72E-05**	3.43E-05	**7.53E-05**
9	**1.60E-05**	**1.10E-04**	**2.47E-05**	1.50E-03	**1.59E-02**
10		1.86E-09	**7.54E-13**	4.32E-02	2.10E-07
11		6.60E-03	9.05E-03	**1.50E-03**	4.69E-01
12			3.21E-04	1.56E-02	3.15E-01
13		**3.03E-07**	3.35E-06	**7.37E-06**	4.53E-01
14		3.54E-03	**2.12E-16**	**2.32E-13**	6.87E-05
15		5.10E-04	1.20E-02	1.10E-04	1.49E-01
16		2.66E-02	8.25E-08	6.64E-05	9.77E-01
17					
18		8.23E-02	4.87E-02		1.53E-01
19		**1.89E-03**			
20			2.05E-04	**3.43E-04**	3.15E-01

LUAD				
Cluster Number	PD-1 Prediction Q-Value	PD-L1 Prediction Q-Value	CTLA-4 Prediction Q-Value	TIGIT Prediction Q-Value
1	6.62E-03	1.86E-04	4.08E-04	**1.62E-03**
2				**1.05E-04**
3	3.67E-02	**6.50E-03**	1.88E-03	2.45E-01
4	**4.77E-04**	4.58E-02	1.07E-04	2.79E-04
5	1.72E-03	1.80E-01	3.25E-03	4.53E-01
6	**7.44E-09**	2.87E-01	1.99E-03	3.46E-01
7	2.06E-01	3.69E-02	6.63E-02	4.53E-01
8	5.73E-02		**3.00E-06**	**1.44E-01**
9	1.46E-03			1.84E-02

10	9.91E-02	1.08E-01	1.96E-02	4.67E-01
11	2.59E-02	2.33E-03	6.01E-01	2.71E-01
12	5.73E-02	4.00E-03	4.89E-04	3.62E-01
13	**3.05E-05**	**1.86E-04**	**3.00E-06**	**1.90E-03**
14	8.88E-01	7.25E-02	2.19E-02	1.03E-01
15	2.79E-01	**4.44E-05**	**3.00E-06**	3.66E-01
16				1.07E-03
17			1.74E-01	1.09E-02
18	**1.51E-04**		**7.95E-03**	4.78E-01
19	**2.43E-03**	**2.33E-03**	**1.89E-03**	**6.67E-03**
20	1.60E-01	**4.23E-08**	5.00E-05	5.57E-02

LUSC				SKCM	
Cluster Number	PD-1 Prediction Q-Value	CTLA-4 Prediction Q-Value	TIGIT Prediction Q-Value	Cluster Number	TIGIT Prediction Q-Value
1	1.30E-01		3.51E-01	1	
2	1.89E-03			2	**2.70E-10**
3	**2.51E-06**	2.88E-01	1.80E-02	3	7.87E-02
4	1.16E-02		2.74E-01	4	1.45E-02
5	4.42E-03		6.70E-01	5	
6	3.30E-02		5.42E-01	6	
7	1.89E-03		4.00E-01	7	
8	1.95E-02	**1.87E-02**	2.11E-02	8	**1.45E-02**
9	7.18E-04	9.13E-01	**5.61E-03**	9	
10	2.03E-02		5.41E-01	10	2.83E-01
11	4.09E-03	2.88E-01	4.66E-02	11	
12	1.21E-02		4.02E-01	12	
13	1.89E-03		3.77E-02	13	
14	**1.46E-02**	**1.49E-01**	**3.73E-02**	14	8.18E-02
15	2.44E-02			15	
16	3.12E-02		2.75E-01	16	
17	3.28E-02		6.70E-01	17	
18	**2.62E-08**		**1.36E-03**	18	
19	2.66E-02			19	**1.28E-06**
20	1.32E-01		**5.61E-03**	20	**2.68E-04**

21	**2.56E-03**	**8.00E-03**	**1.36E-03**	21	
22	5.96E-02			22	**5.19E-04**
23	**1.33E-04**			23	
24	9.96E-02	**1.33E-02**	8.27E-03	24	
25	1.07E-01	**8.00E-03**			

STAD					
Cluster Number	PD-1 Prediction Q-Value	PD-L1 Prediction Q-Value	CTLA-4 Prediction Q-Value	HRD Prediction Q-Value	TIGIT Prediction Q-Value
1	1.47E-01	2.65E-05	**2.13E-03**	5.93E-02	1.28E-03
2	7.90E-01	**7.26E-06**	9.76E-02	**1.50E-05**	4.69E-04
3	1.47E-01	4.89E-04	6.43E-03		8.40E-04
4	7.51E-01	2.40E-04	1.24E-02		4.40E-02
5		7.60E-08	1.86E-02	**1.50E-05**	3.24E-04
6		1.88E-07	6.20E-07	5.85E-02	3.24E-04
7		**7.19E-05**	7.16E-01		5.76E-04
8		5.95E-06	2.49E-01		2.18E-03
9	**2.78E-03**	8.27E-04	7.09E-01		2.99E-02
10	**4.81E-03**	8.27E-04	**1.04E-03**	5.85E-02	6.19E-03
11		**7.47E-07**	**6.51E-05**		2.60E-03
12		5.93E-07	**1.63E-05**		8.80E-05
13	8.42E-02	1.21E-05	**1.04E-03**	4.55E-02	3.87E-03
14		9.15E-05	6.72E-02		**5.05E-04**
15		**9.55E-09**	3.46E-05	1.20E-01	2.04E-04
16	**7.00E-06**	2.37E-06	1.05E-03		**5.00E-06**
17		3.21E-01	1.18E-05	**2.05E-02**	1.03E-01
18					1.89E-01
19		2.72E-03	1.71E-02		1.52E-01
20	**1.40E-03**	**5.37E-09**	6.24E-02	**5.28E-03**	**2.04E-04**
21		7.35E-03	4.18E-03		**3.24E-04**
22	1.83E-02	1.73E-15	1.22E-07	**1.48E-02**	**1.70E-05**
23	**2.78E-03**		1.24E-02		8.63E-02

Supplemental Figure 1. Cell- and tissue-level heatmap examples by cancer type
Unprocessed portions of BRCA, LUAD, LUSC, and SKCM H&E-stained slides alongside corresponding overlays (heatmaps) of cell- and tissue-type predictions. Slide regions are classified into tissue types: cancer tissue (red), cancer-associated stroma (orange), necrosis (black), or normal (transparent). Pixels in cancer tissue or cancer-associated stroma areas are classified into cell types: lymphocyte (green), plasma cell (lime), fibroblast (orange), macrophage (aqua), cancer cell (red), or background (transparent). Examples for STAD are shown in Figure 1c.

BRCA

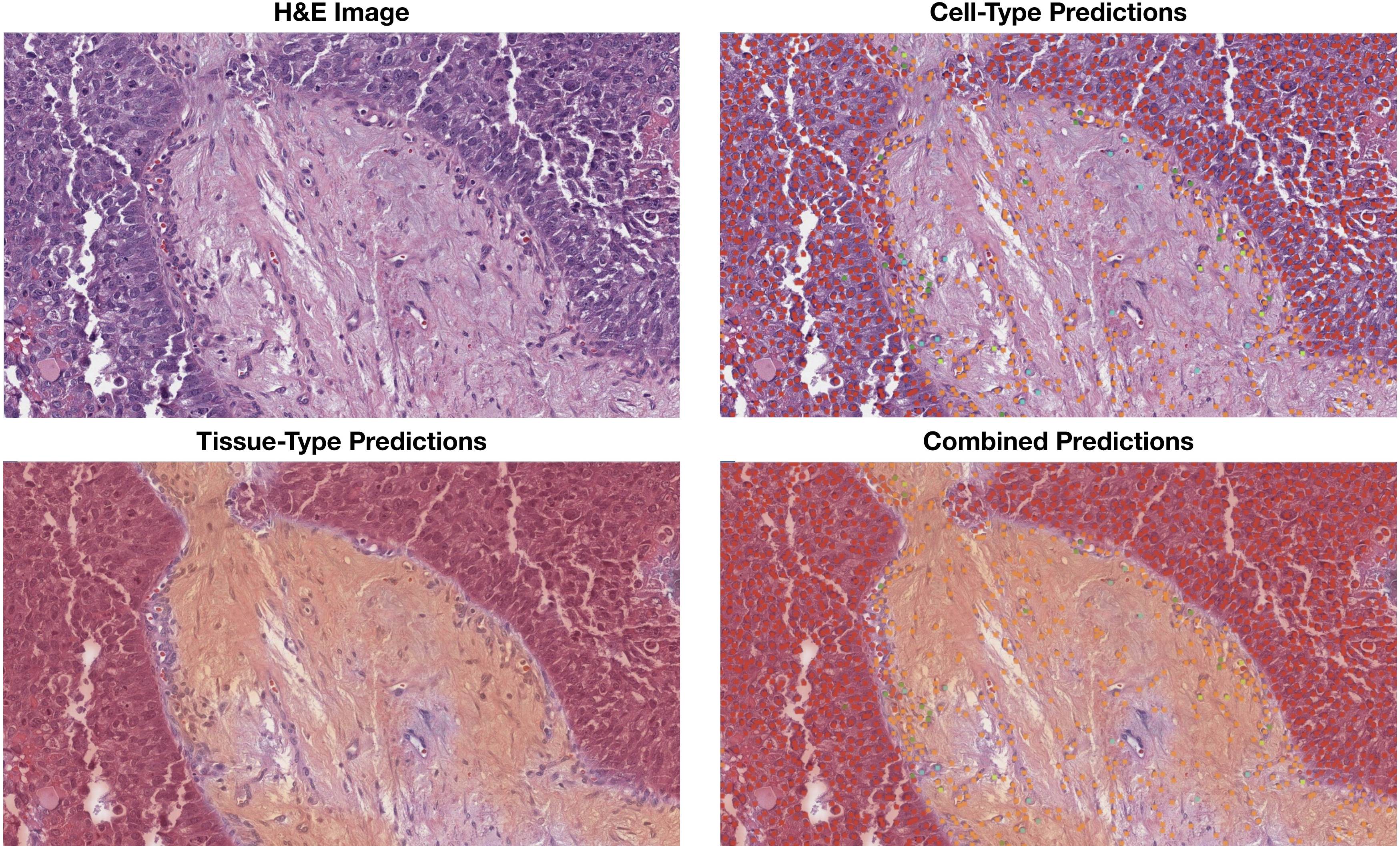

1.1 mm x 600 μm (Width x Height)

LUAD

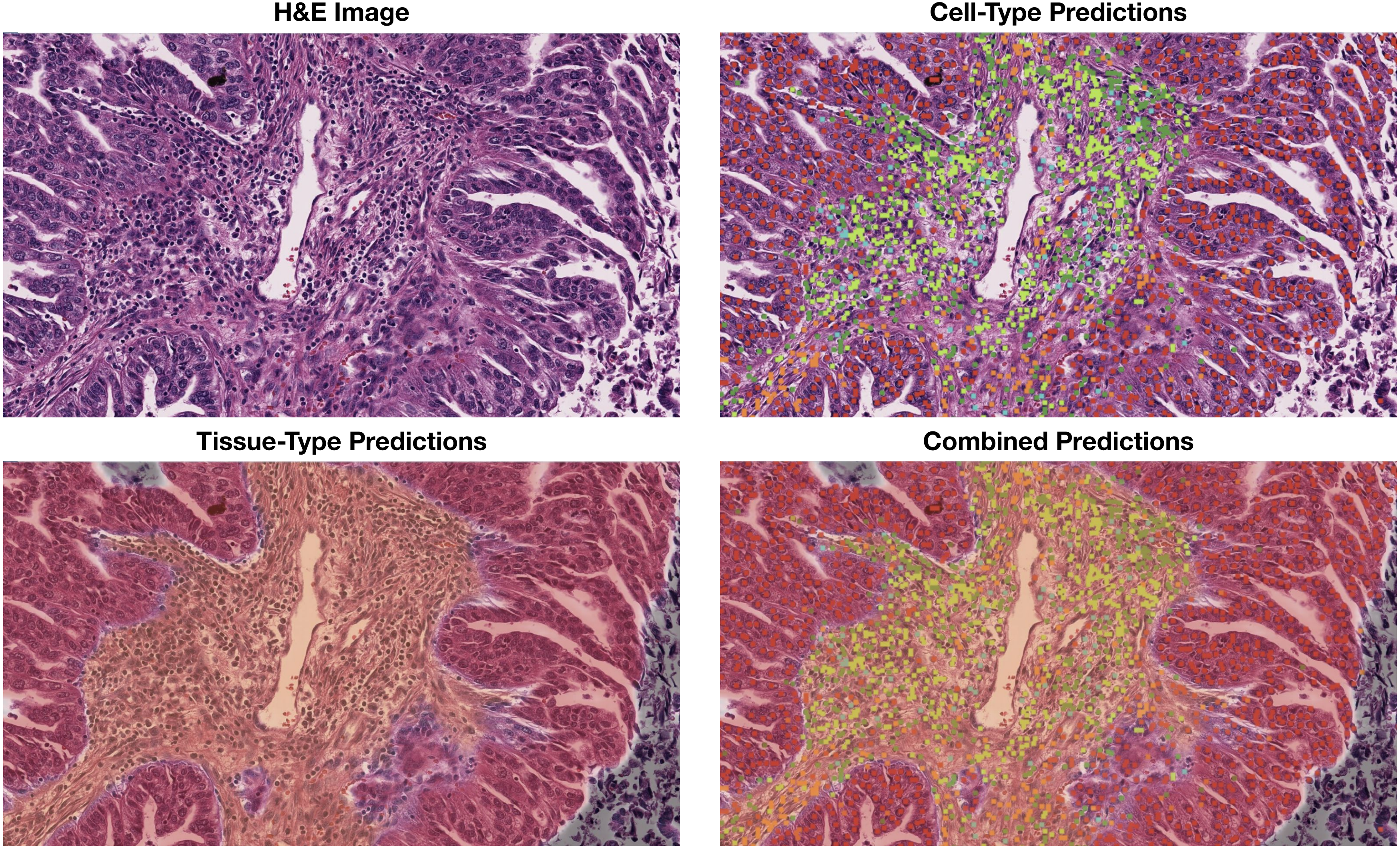

1.1 mm x 600 µm (Width x Height)

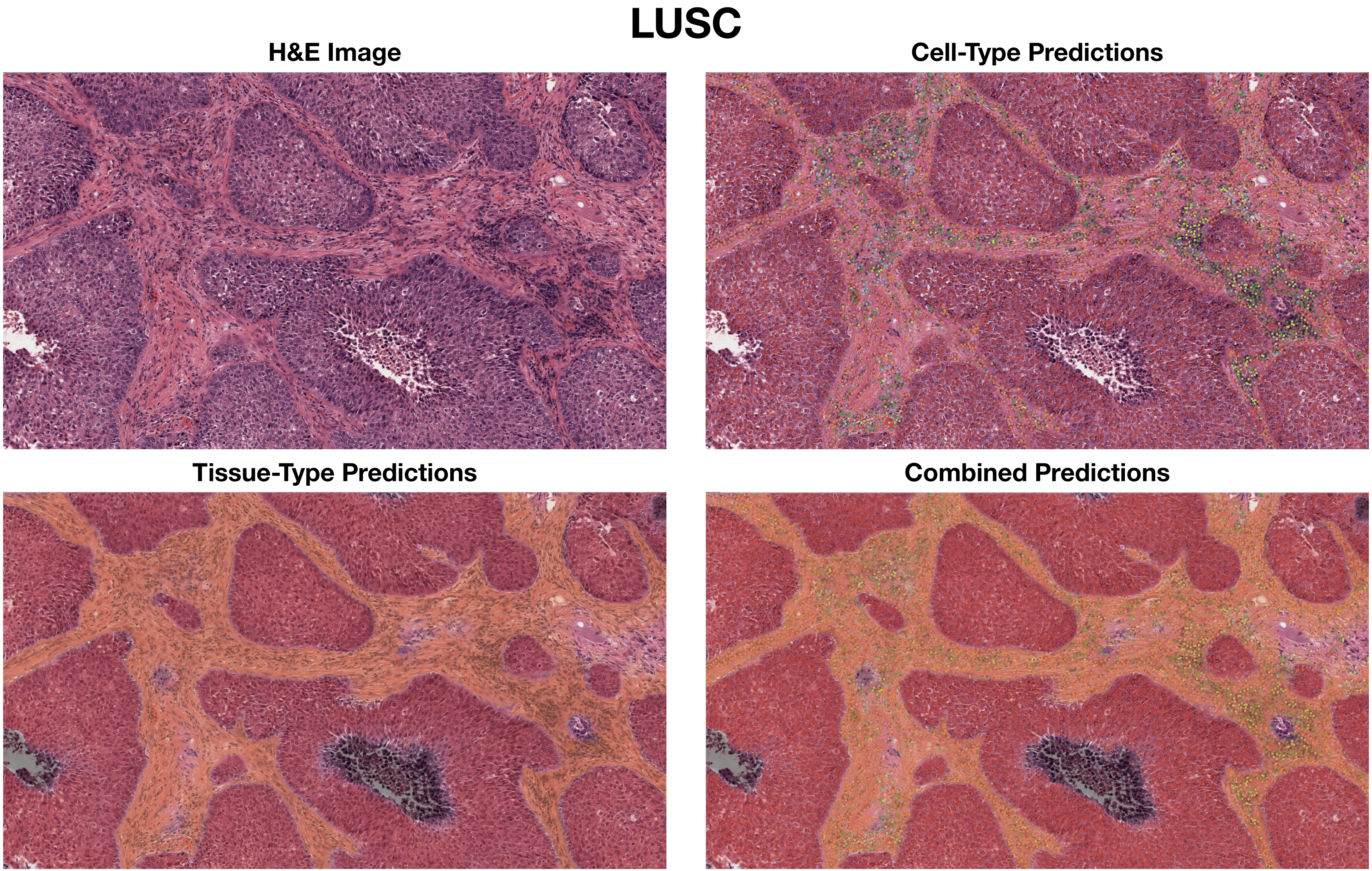

2.5 mm x 1.4 mm (Width x Height)

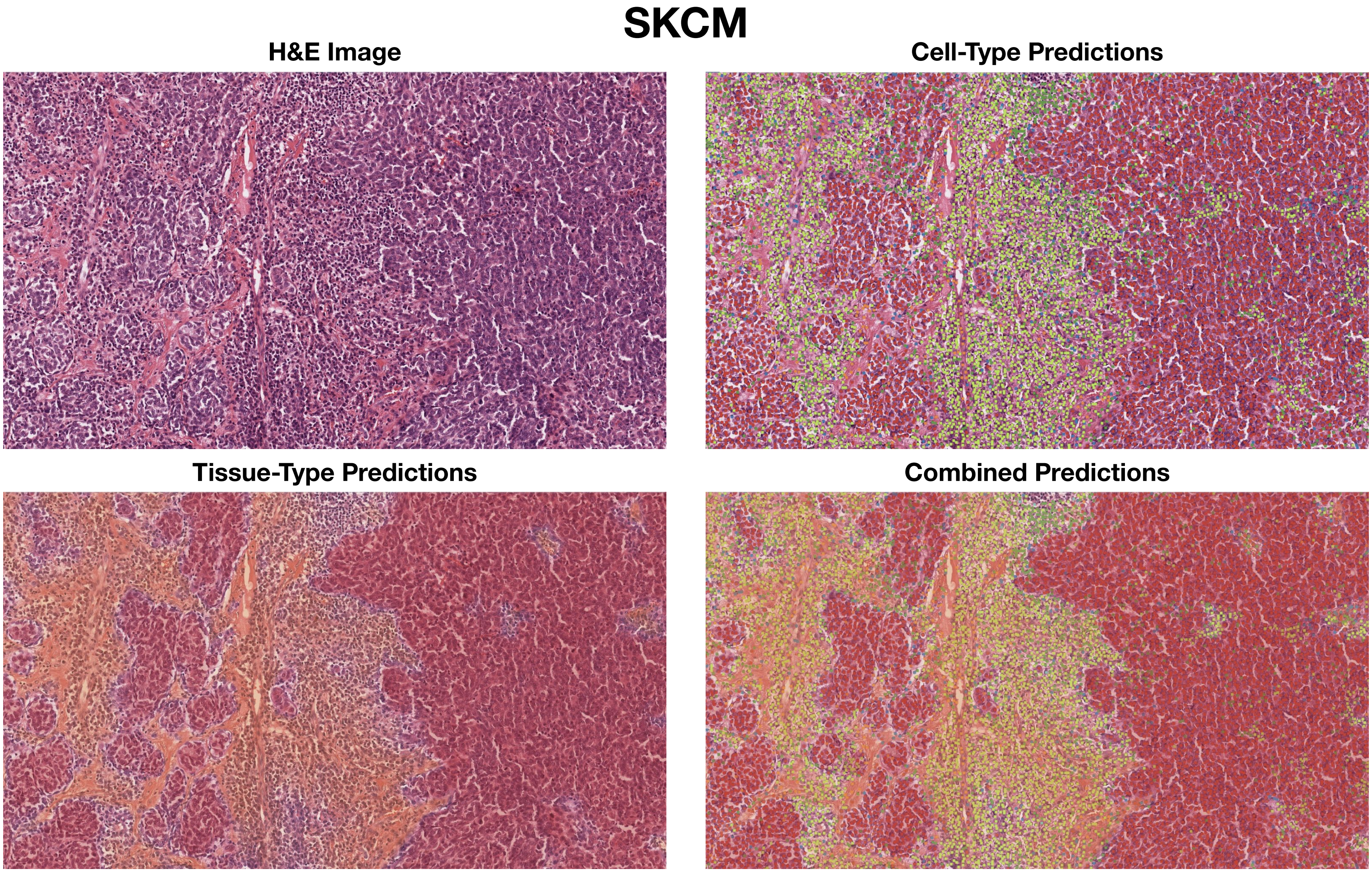

1.9 mm x 1.0 mm (Width x Height)

Supplemental Figure 2. Cell- and tissue-type model validation accuracies
Confusion matrices showing accuracy metrics of cell- and tissue-type models evaluated on pooled primary and secondary annotations in the validation set. The ground truth consists of pathologist annotations of WSIs in the validation set. Cell-type confusion matrices show accuracy metrics for background (B), lymphocytes (L), plasma cells (PC), fibroblasts (F), macrophages (M), and cancer (epithelial) cells (CEC). Tissue-type confusion matrices show accuracy metrics for normal tissue (N), cancer tissue (C), necrosis (N), and cancer-associated stroma (CAS).

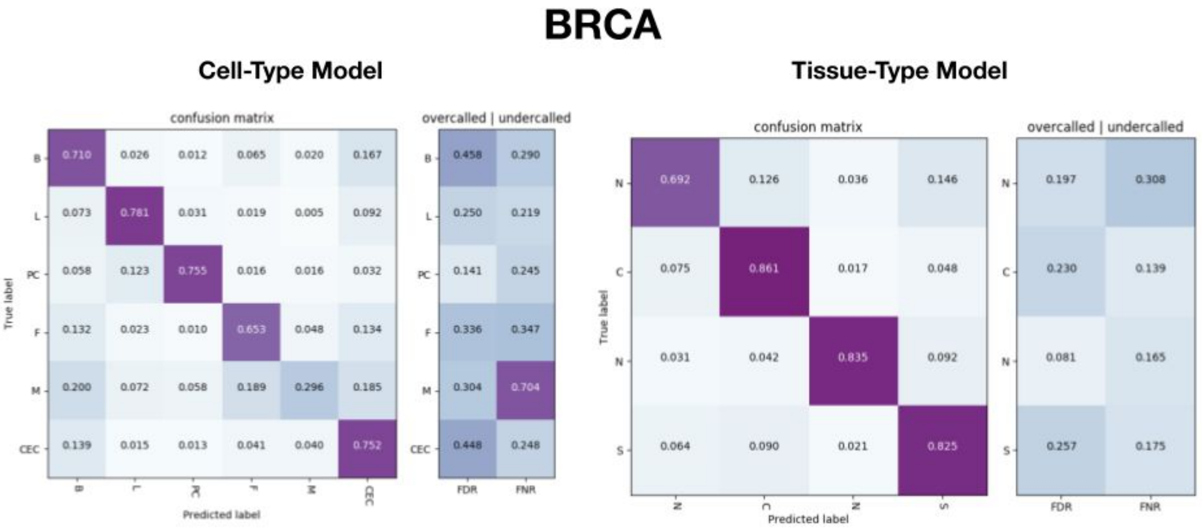

LUAD

Cell-Type Model

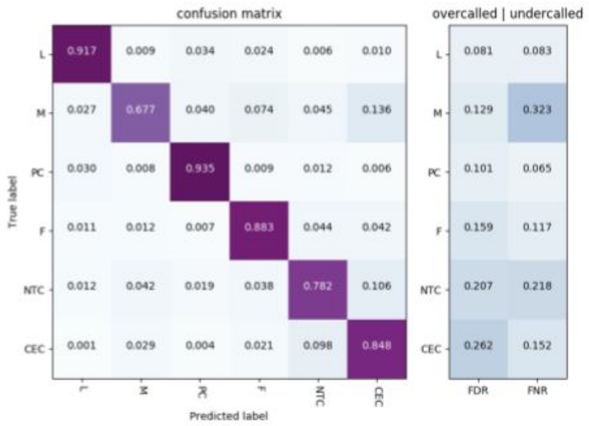

Tissue-Type Model

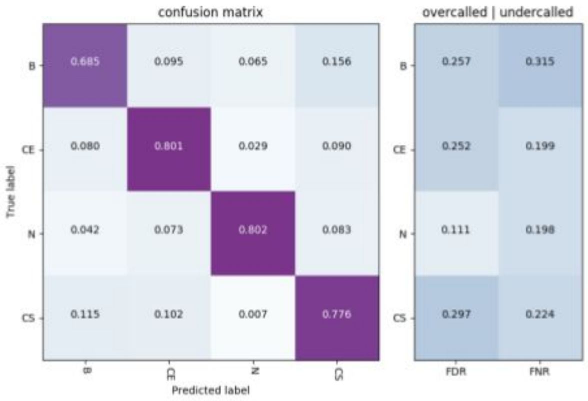

LUSC

Cell-Type Model

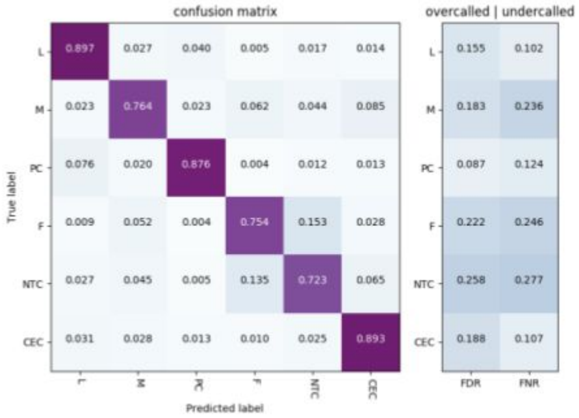

Tissue-Type Model

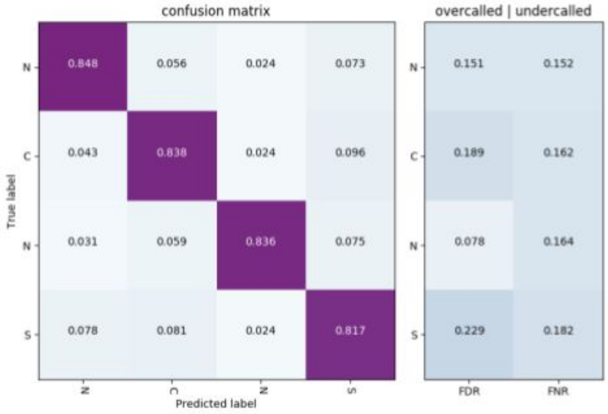

STAD

Cell-Type Model

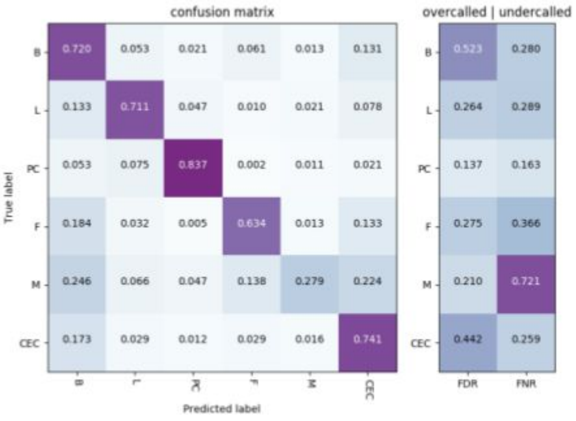

Tissue-Type Model

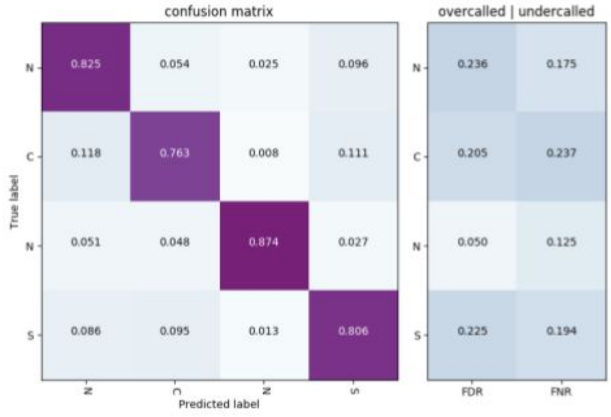

SKCM

Cell-Type Model

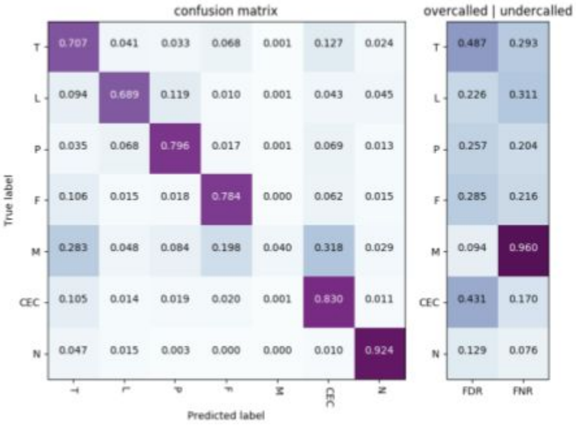

Tissue-Type Model

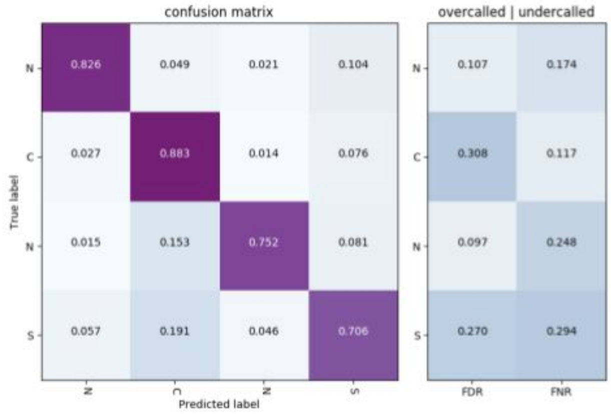

Supplemental Figure 3. Frames validation of cell-type model predictions against pathologist cell-type annotations

We generated 250 75x75 μm frames of cell-type overlays evenly sampled across the five cancer types and five cell types, each from a distinct WSI. Each frame was annotated for each of the five cell types by five board-certified pathologists. This allows us to compare the count of lymphocytes, plasma cells, fibroblasts, macrophages, and cancer cells in each 75x75 μm frame predicted by our CNN cell-type models against a consensus of pathologist annotation counts. We computed the Pearson correlation between our cell-type model counts and pathologist consensus counts across the 250 frames for all five cell types. Pathologist consensus counts were computed as the median of the five individual pathologist counts for a given frame and cell type. To capture inter-pathologist variability, we also computed the leave-one-out Pearson correlation between each individual pathologist's annotation counts and the consensus (median) among the remaining four pathologists. We then obtained a point estimate and 95% confidence interval for the average performance of an annotator with respect to the leave-one-out consensus. We observe robust correlations between cell-type model predictions and pathologist consensi for cancer cells, lymphocytes, and plasma cells. While the absolute correlation observed for macrophages and fibroblasts is lower, such correlations are on par with inter-pathologist variability.

Cell Type	Model vs. Pathologist Consensus Correlation (95% CI)	Inter-Pathologist Correlation (95% CI)
Cancer Cells	0.811 (0.763, 0.849)	0.880 (0.832, 0.914)
Lymphocytes	0.904 (0.879, 0.925)	0.880 (0.830, 0.916)
Plasma Cells	0.681 (0.609, 0.743)	0.700 (0.527, 0.811)
Macrophages	0.392 (0.282, 0.492)	0.465 (0.391, 0.532)
Fibroblasts	0.511 (0.413, 0.597)	0.618 (0.552, 0.676)

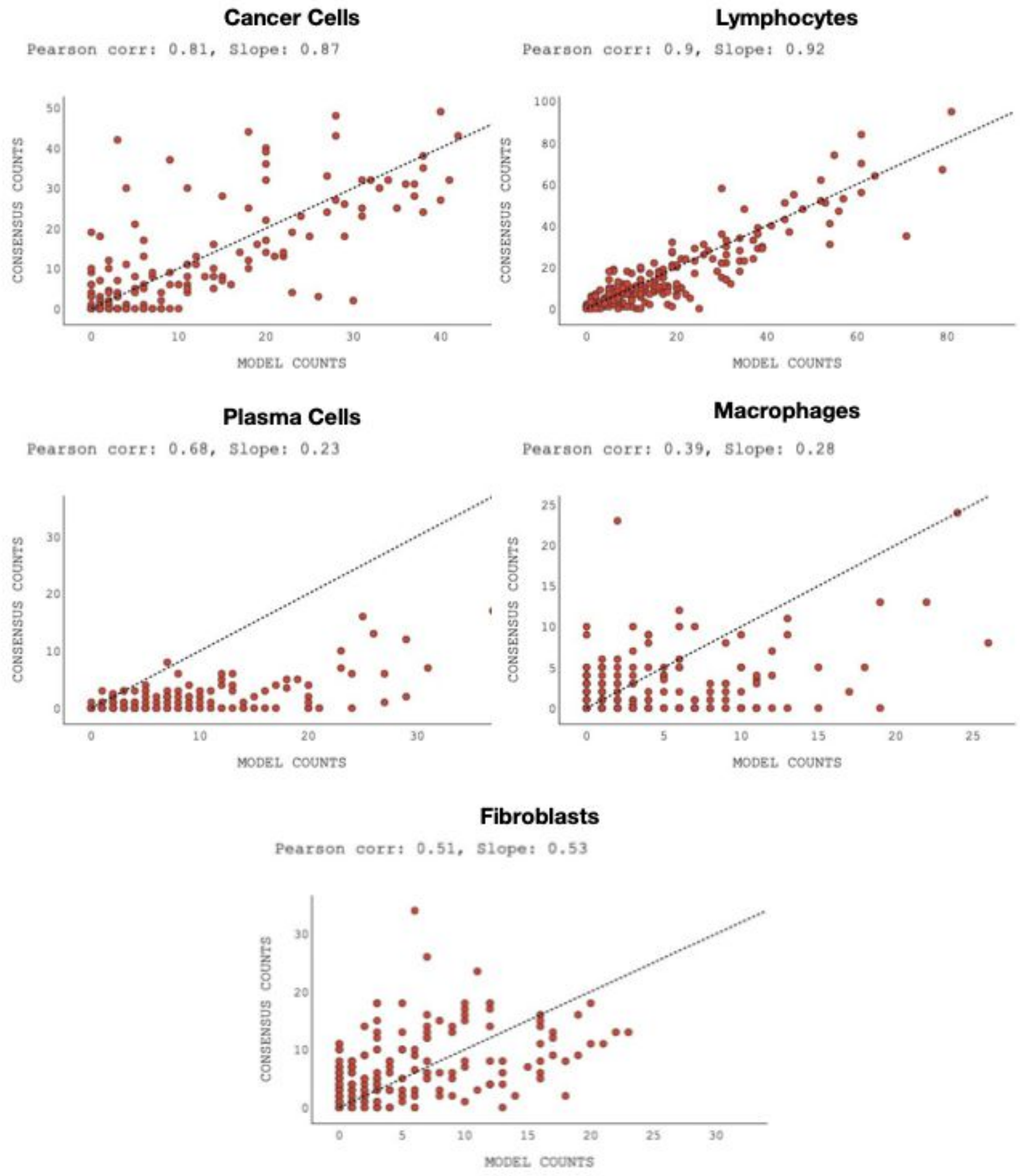
Cancer Cells
Pearson corr: 0.81, Slope: 0.87
CONSENSUS COUNTS
MODEL COUNTS
Lymphocytes
Pearson corr: 0.9, Slope: 0.92
CONSENSUS COUNTS
MODEL COUNTS
Plasma Cells
Pearson corr: 0.68, Slope: 0.23
CONSENSUS COUNTS
MODEL COUNTS
Macrophages
Pearson corr: 0.39, Slope: 0.28
CONSENSUS COUNTS
MODEL COUNTS
Fibroblasts
Pearson corr: 0.51, Slope: 0.53
CONSENSUS COUNTS
MODEL COUNTS

Supplemental Figure 4. Cell-type model external validation

To assess model generalizability, we redeployed our BRCA cell-type model trained primarily on TCGA to exhaustively predict cell types on 72 H&E, FFPE WSIs from an external BRCA dataset uploaded by Peikari et al. to The Cancer Imaging Archive (TCIA). We then used the same analysis framework and metrics described in Supplemental Figure 3 to assess concordance between our cell-type model and pathologist consensus across 250 75x75 μm frames (evenly sampled across the five cell types) generated from these external WSIs.

Cell Type	Model vs. Pathologist Consensus Correlation (95% CI)	Inter-Pathologist Correlation (95% CI)
Cancer Cells	0.894 (0.866, 0.916)	0.970 (0.960, 0.978)
Lymphocytes	0.926 (0.906, 0.942)	0.928 (0.904, 0.946)
Plasma Cells	0.753 (0.802, 0.842)	0.880 (0.819, 0.922)
Macrophages	0.607 (0.522, 0.680)	0.583 (0.496, 0.658)
Fibroblasts	0.726 (0.661, 0.780)	0.650 (0.575, 0.714)

Cancer Cells

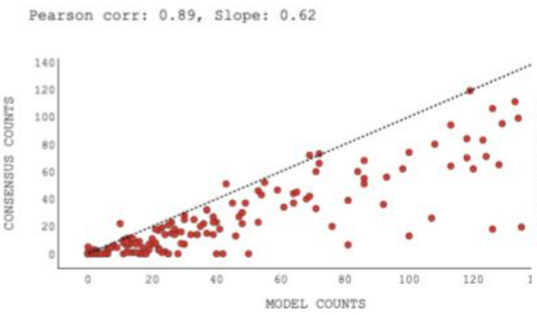

Lymphocytes

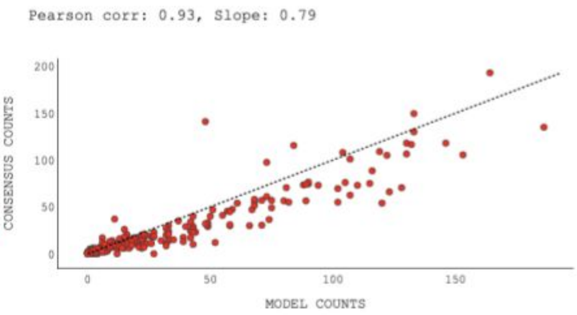

Plasma Cells

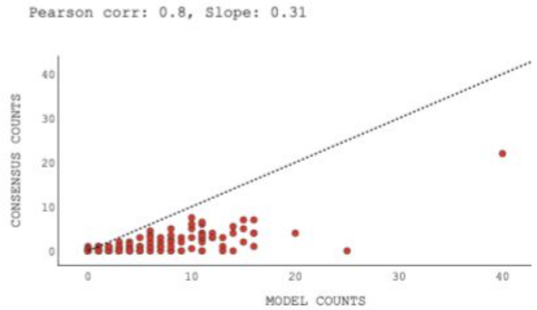

Macrophages

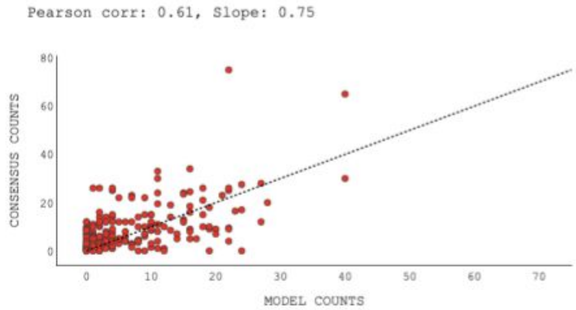

Fibroblasts

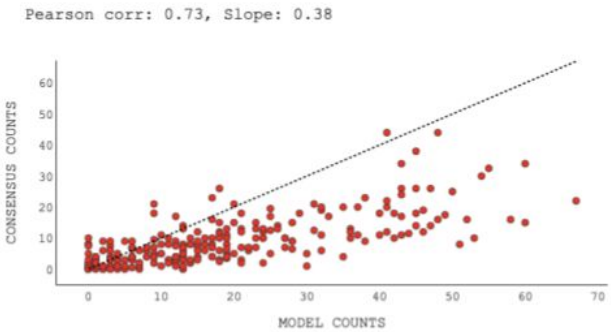

Supplemental Figure 5. SKCM-stratified analyses

SKCM metastasis versus primary samples were inferred using the TCGA sample code ("06" versus "01," respectively). HIF differences between SKCM metastasis and primary samples are shown in the context of the four other cancer types. Although the SKCM subtypes are quite similar in relation to other cancer types, several differences emerge: the average solidity of significant regions of CT+CAS, area of CT, and density of fibroblasts in CAS were greater in metastasis samples, while the average eccentricity of significant regions of necrotic tissue and density of cancer cells in CT were greater in primary samples. Sparse-group lasso models were trained on SKCM metastasis samples (n=256) to predict TIGIT binary labels using the same hyper-parameters identified during un-stratified SKCM-TIGIT model training. The metastasis-trained model was then evaluated on SKCM primary samples (n=62), with the resultant ROC and precision-recall curves shown below.

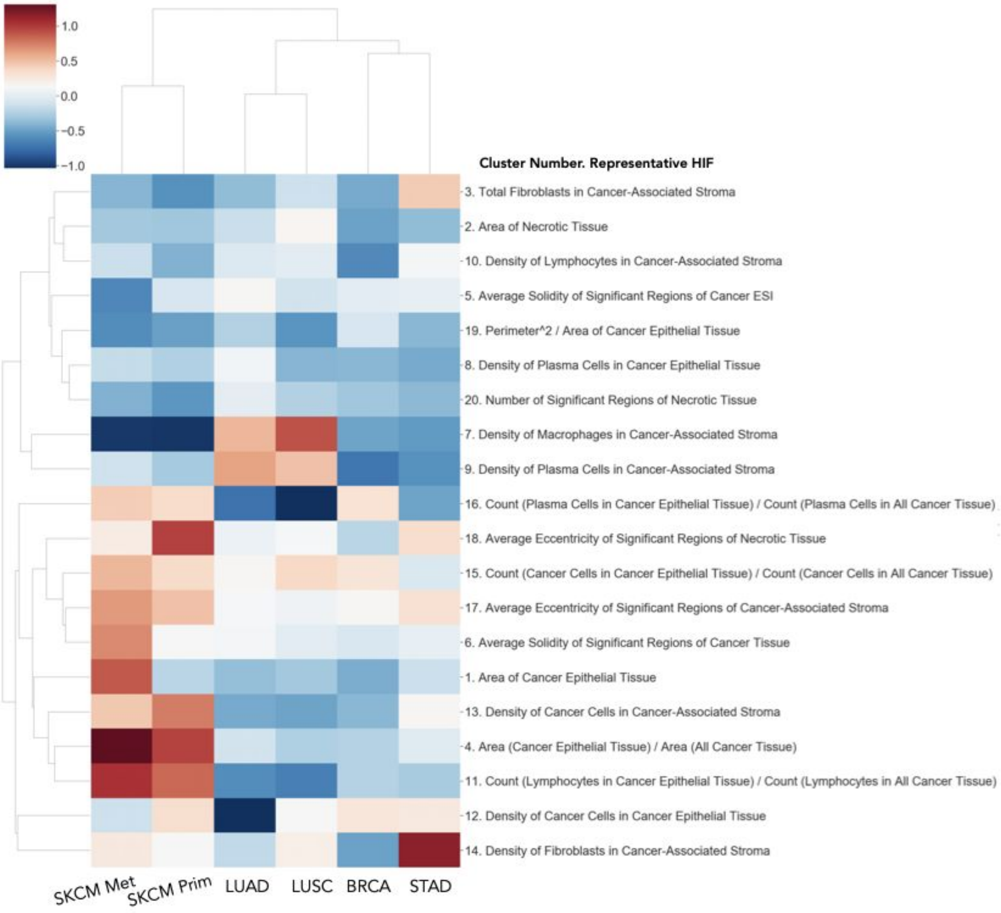

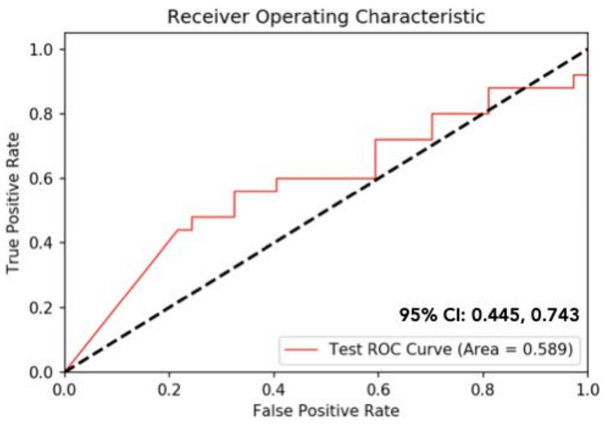
Receiver Operating Characteristic
True Positive Rate
False Positive Rate
95% CI: 0.445, 0.743
Test ROC Curve (Area = 0.589)
0.0
0.2
0.4
0.6
0.8
1.0

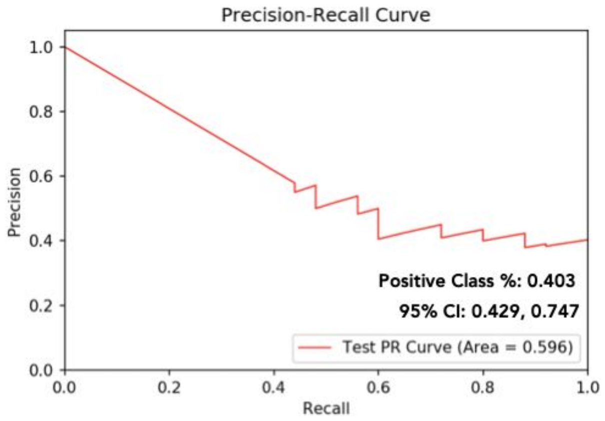
Precision-Recall Curve
Precision
Recall
Positive Class %: 0.403
95% CI: 0.429, 0.747
Test PR Curve (Area = 0.596)
0.0
0.2
0.4
0.6
0.8
1.0

Supplemental Figure 6. Spatial heterogeneity around cancer-stroma interface
Heatmap exhibiting the density ratio of each of the five cell types near the cancer-stroma interface relative to in cancer tissue and cancer-associated stroma broadly, stratified across the five cancer types. At the CSI, SKCM has an enrichment of lymphocytes and plasma cells, LUSC has an enrichment of cancer cells and a reduction of plasma cells, LUAD has an enrichment of cancer cells, and BRCA has the lowest density of macrophages.

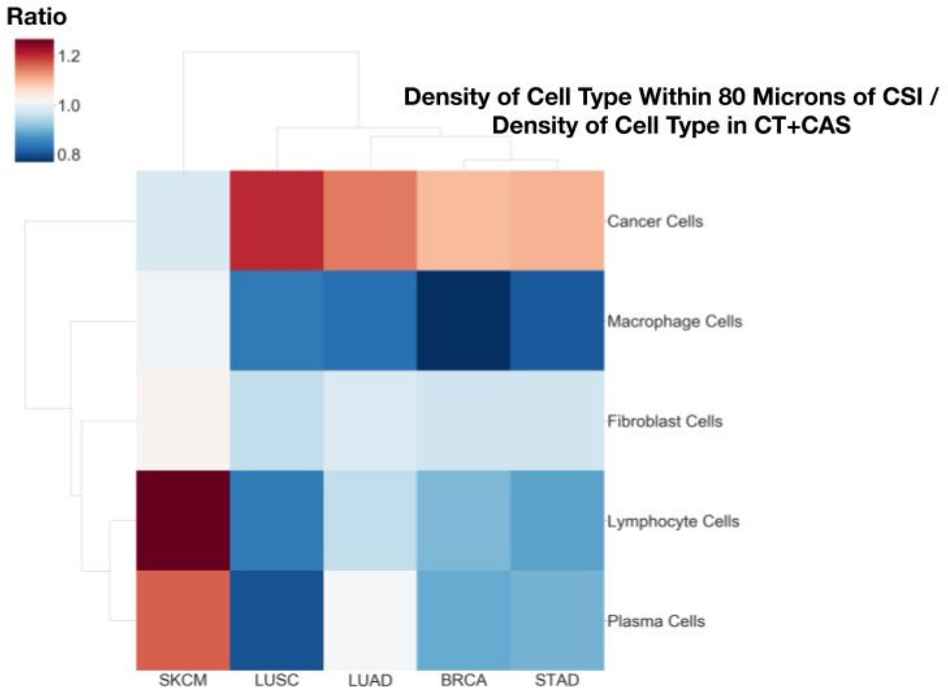

Supplemental Figure 7. Validation of HIFs against molecular deconvolution of immune cell contributions

Comparison of leukocyte fraction, plasma cell fraction, and lymphocyte fraction estimates from our 2-micron resolution cell-type model predictions (x-axis) and CIBERSORT RNA-Seq deconvolutions (y-axis) across all patient samples. All measured Spearman correlations (0.40-0.55) are positive and consistent with informative image-based features. Potential sources of disagreement include RNA contributions from tumor, fibroblast, and other non-immune cells, discrepancies between 3D bulk samples and 2D tissue slices, tissue sampling variability, and RNA-Seq measurement variability. P-value computations were two-sided; exact P-values were not provided since 2.2E-16 is the smallest positive double-precision floating point value represented in R version 3.6.2.

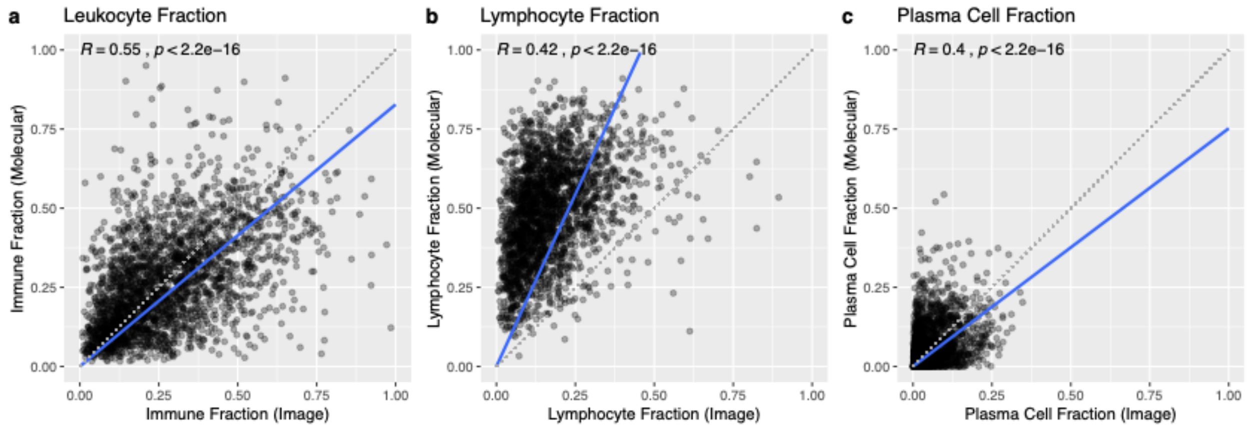

Supplemental Figure 8. Correlation analysis for angiogenesis and hypoxia signatures

Correlation and kernel density estimation plots between representative HIFs and angiogenesis / hypoxia signature scores. Points are colored by cancer type. Note that the X-axis is log-transformed (base ten) in the angiogenesis correlation plot. Error bands represent the 95% confidence interval of the slope line.

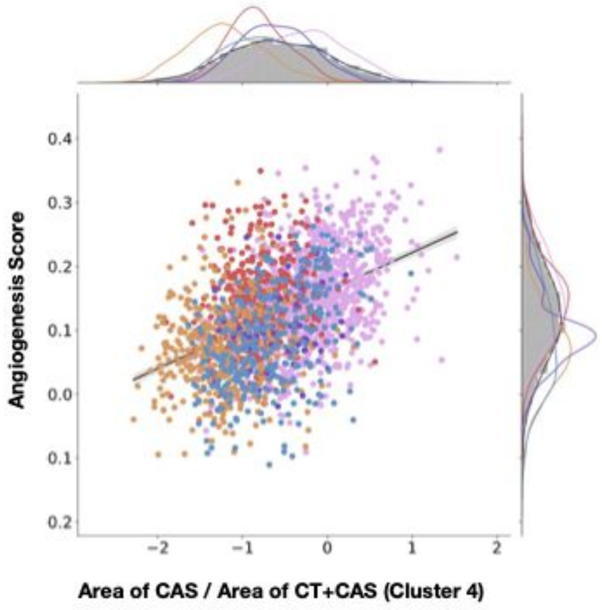

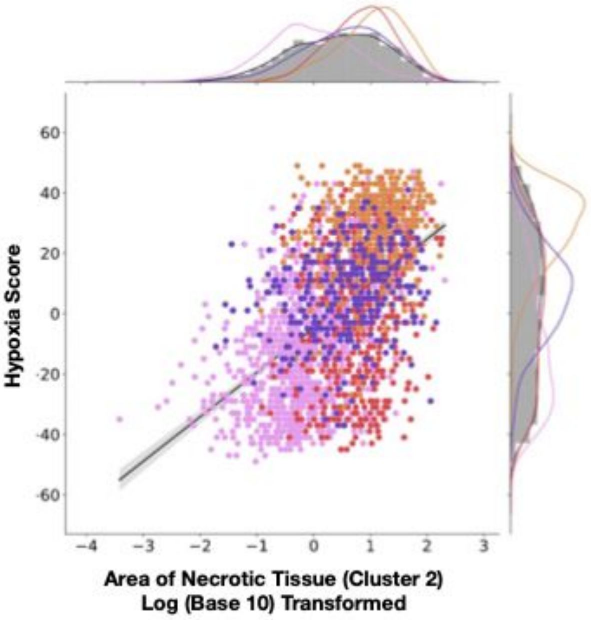

Supplemental Figure 9. Gaussian mixture model thresholding
Histogram of (a) PD-1 expression, (b) PD-L1 expression, (c) CTLA-4 expression, (d) HRD score, and (e) TIGIT expression broken down by cancer type. Continuous immune checkpoint protein expression and HRD scores were binarized to high versus low classes using gaussian mixture model (GMM) clustering with unequal variance. The binary thresholds annotated below were defined as the intersection of the empirical densities between the two GMM-defined clusters. The number and percentage of positive labels per outcome are shown.

a)

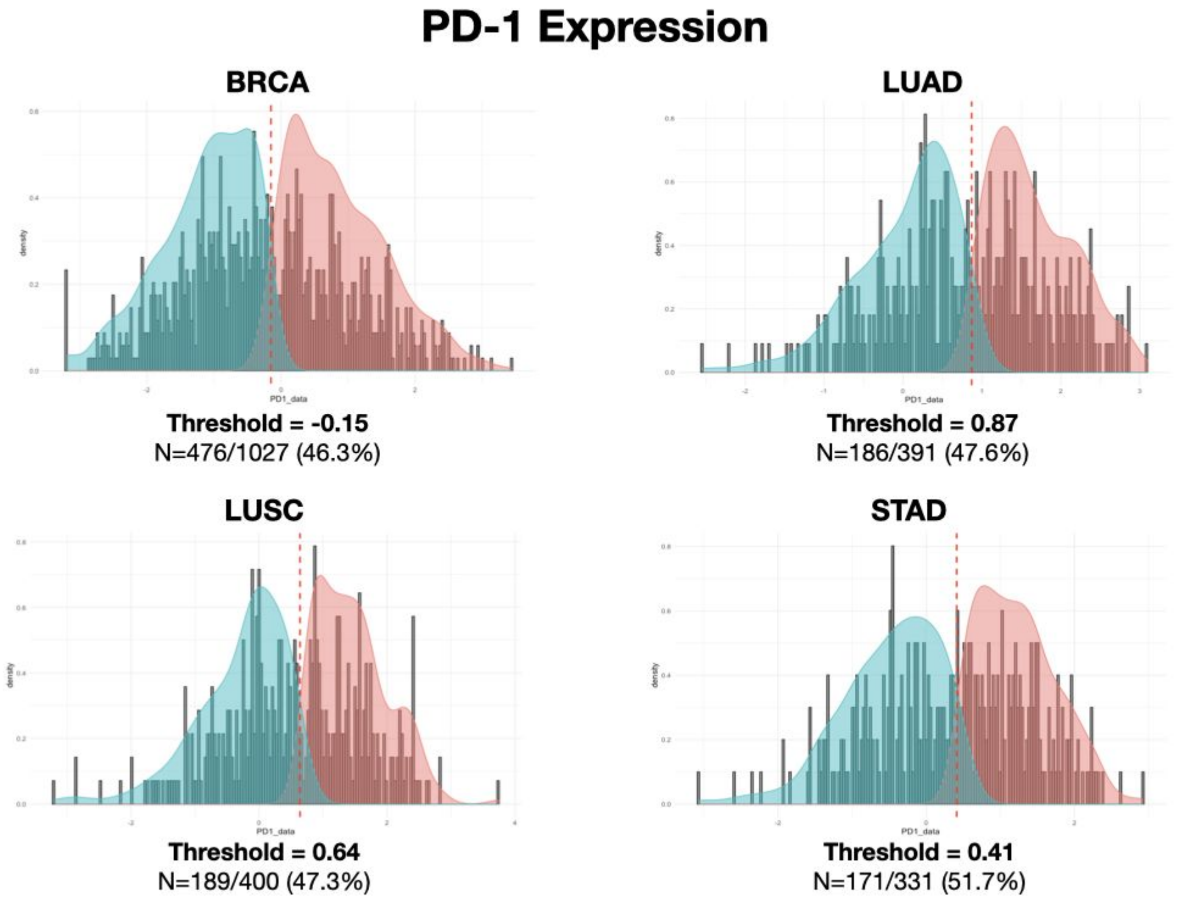

b)

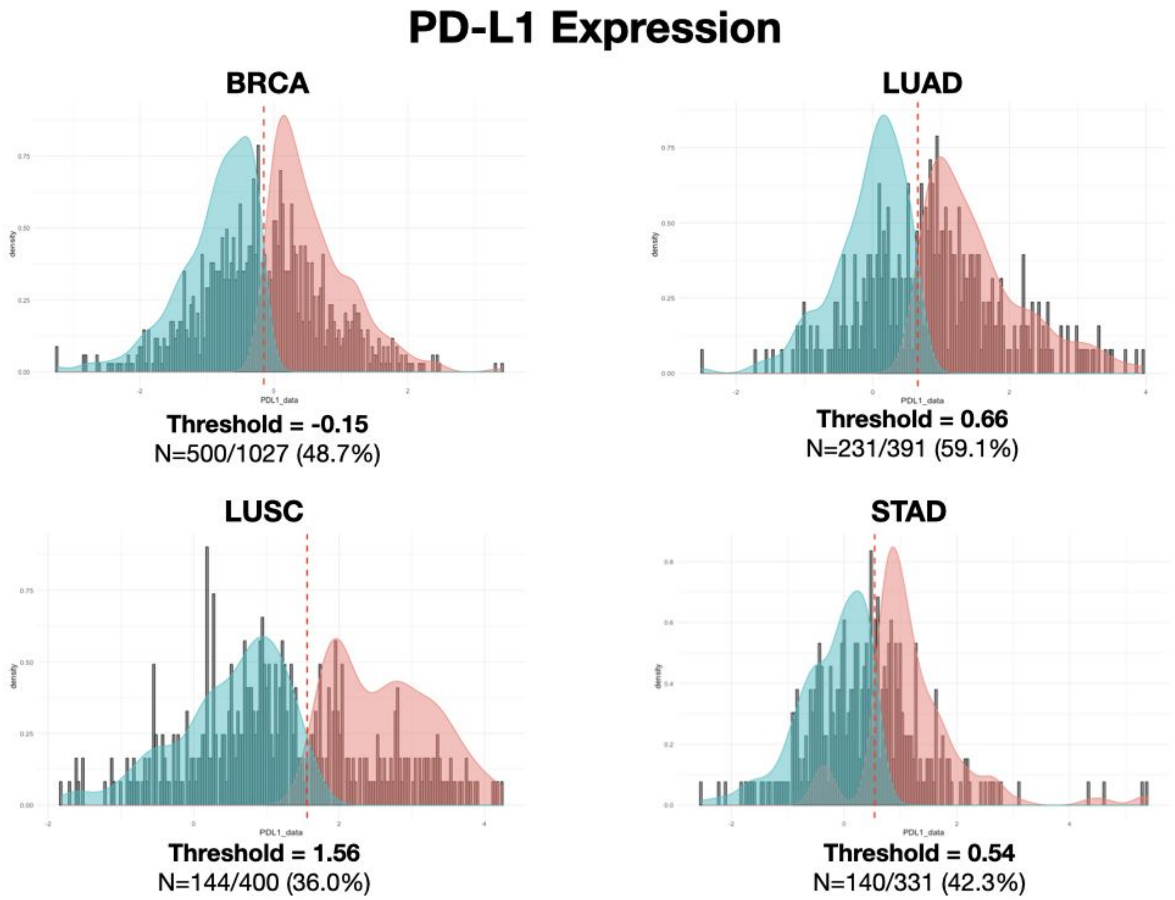
PD-L1 Expression
BRCA
Threshold = -0.15
N=500/1027 (48.7%)
LUAD
Threshold = 0.66
N=231/391 (59.1%)
LUSC
Threshold = 1.56
N=144/400 (36.0%)
STAD
Threshold = 0.54
N=140/331 (42.3%)

c)

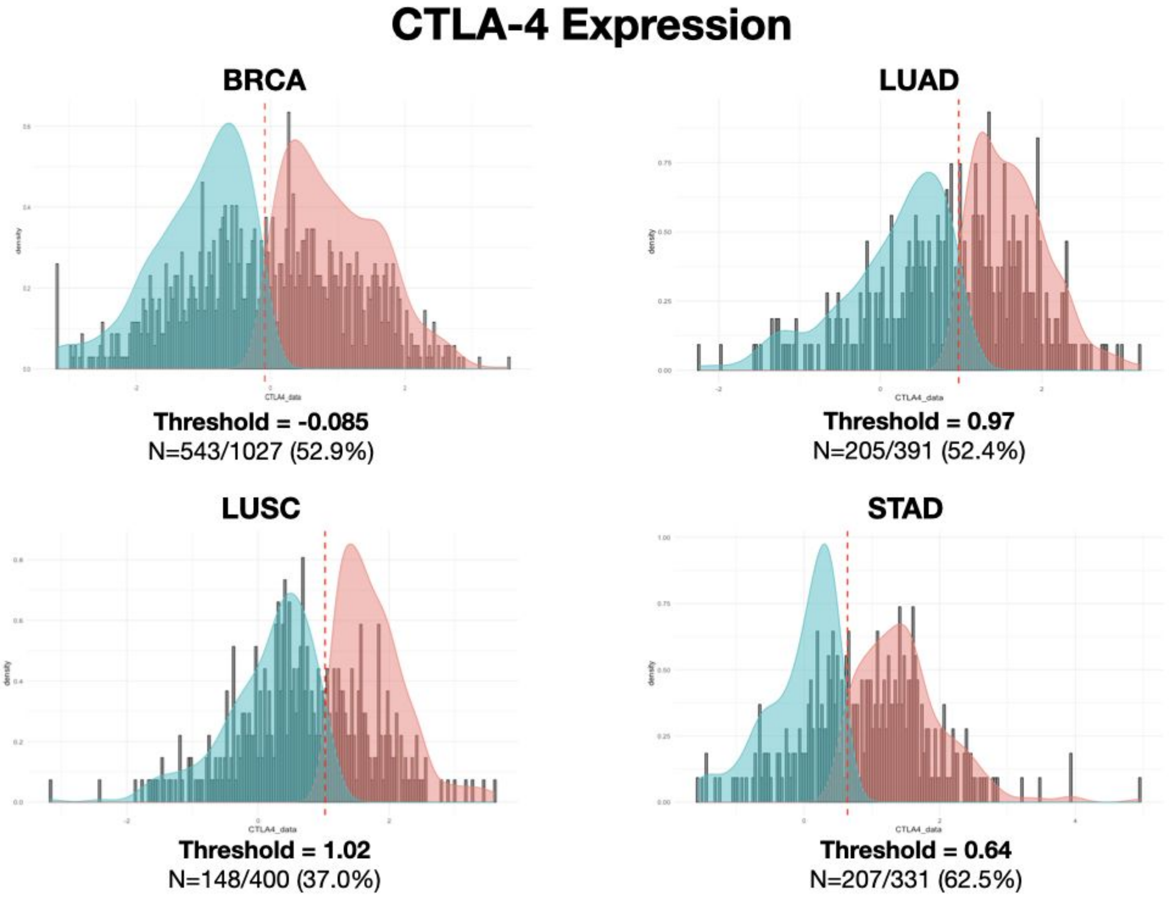
CTLA-4 Expression
BRCA
Threshold = -0.085
N=543/1027 (52.9%)
LUAD
Threshold = 0.97
N=205/391 (52.4%)
LUSC
Threshold = 1.02
N=148/400 (37.0%)
STAD
Threshold = 0.64
N=207/331 (62.5%)

d)

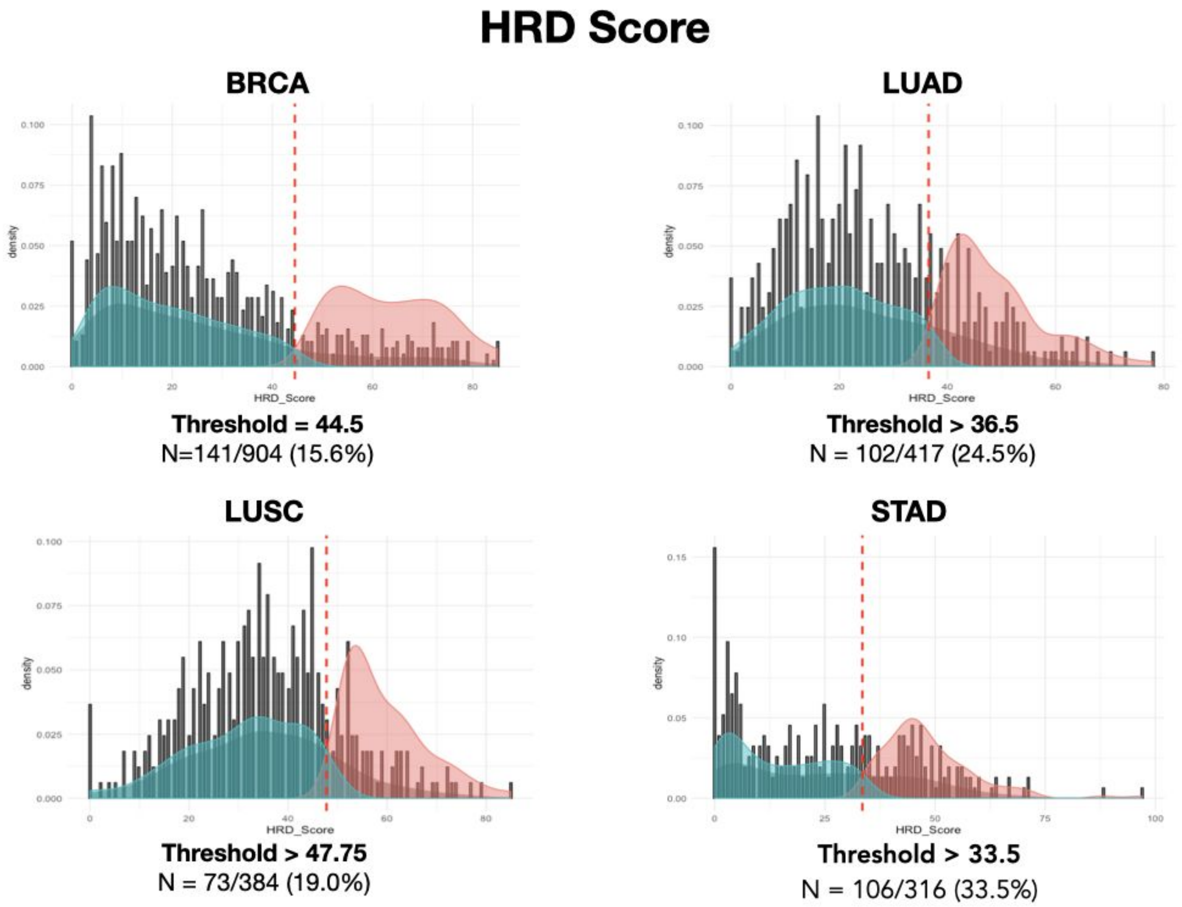
HRD Score
BRCA
LUAD
density
HRD_Score
Threshold = 44.5
N=141/904 (15.6%)
Threshold > 36.5
N = 102/417 (24.5%)
LUSC
STAD
Threshold > 47.75
N = 73/384 (19.0%)
Threshold > 33.5
N = 106/316 (33.5%)

e)

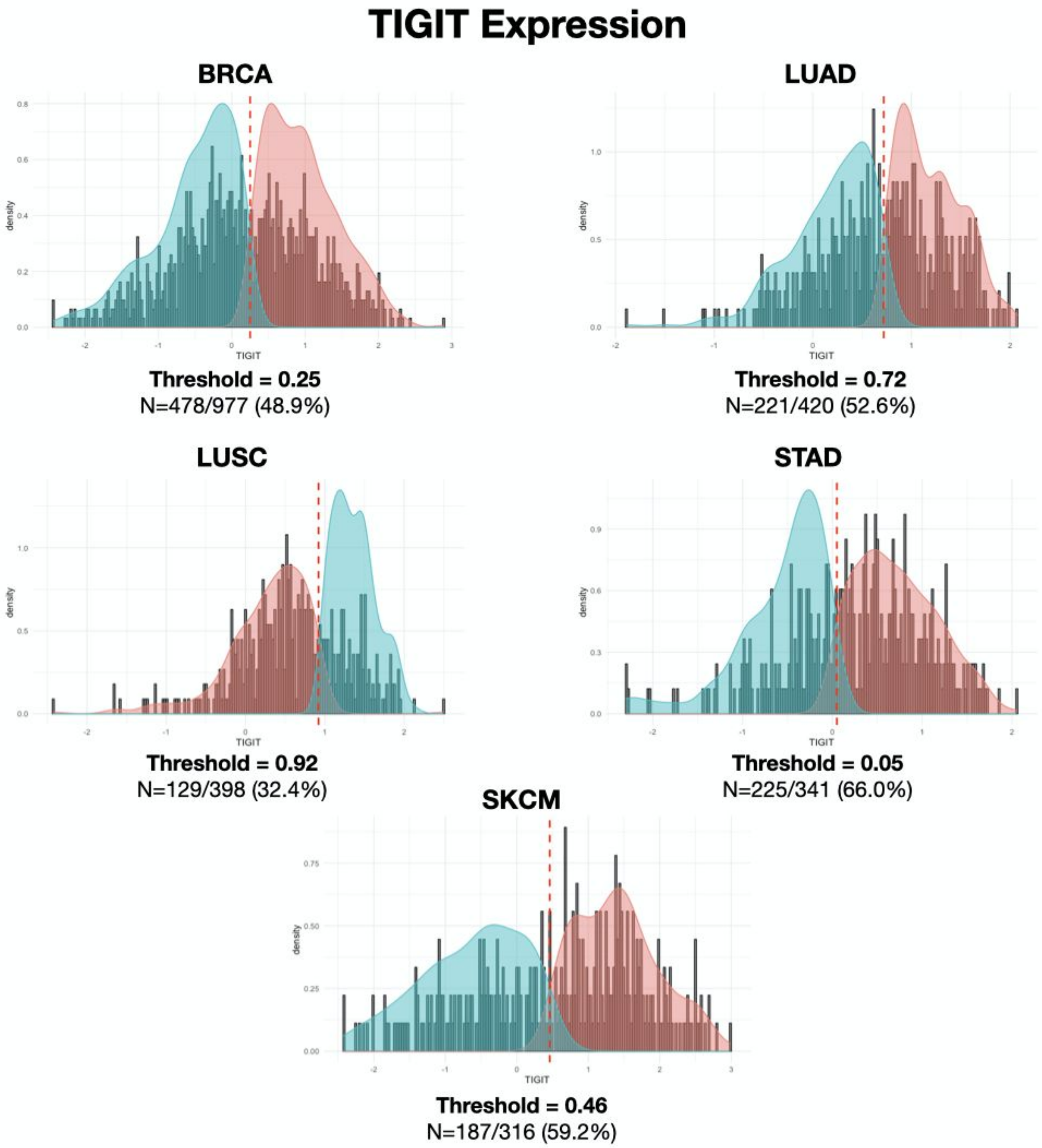
TIGIT Expression
BRCA
Threshold = 0.25
N=478/977 (48.9%)
LUAD
Threshold = 0.72
N=221/420 (52.6%)
LUSC
Threshold = 0.92
N=129/398 (32.4%)
STAD
Threshold = 0.05
N=225/341 (66.0%)
SKCM
Threshold = 0.46
N=187/316 (59.2%)
density
TIGIT

Supplemental Figure 10. Precision-recall curves from prediction of molecular phenotypes using HIFs

Precision-recall (PR) curves for hold-out predictions, colored by cancer type. A random classifier corresponds to an AUPRC equal to the percentage of positive labels for a given prediction task (i.e. a horizontal PR curve drawn at the said percentage). Pan-cancer in TIGIT predictions encompasses all five cancer types. Pan-cancer for the remainder of prediction tasks excluded SKCM due to insufficient outcome labels.

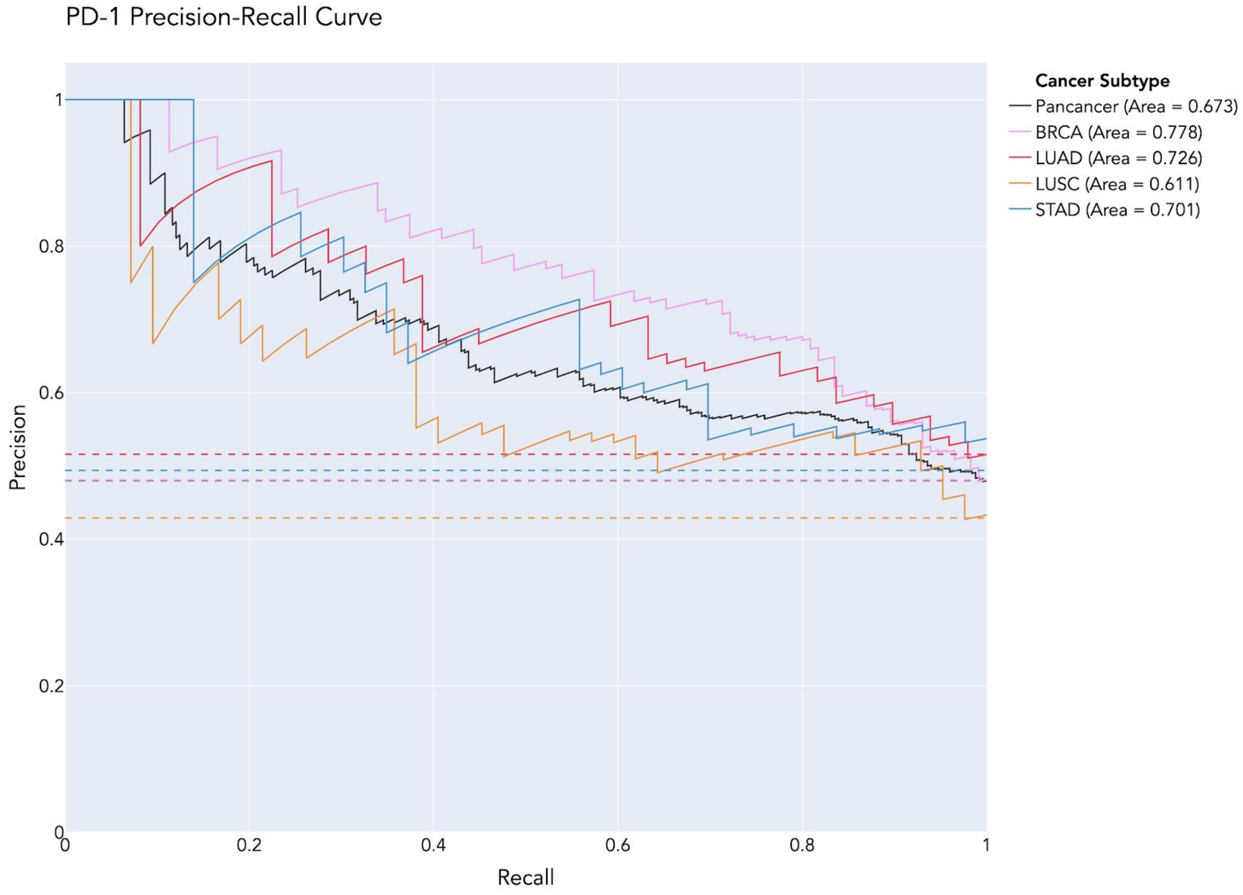

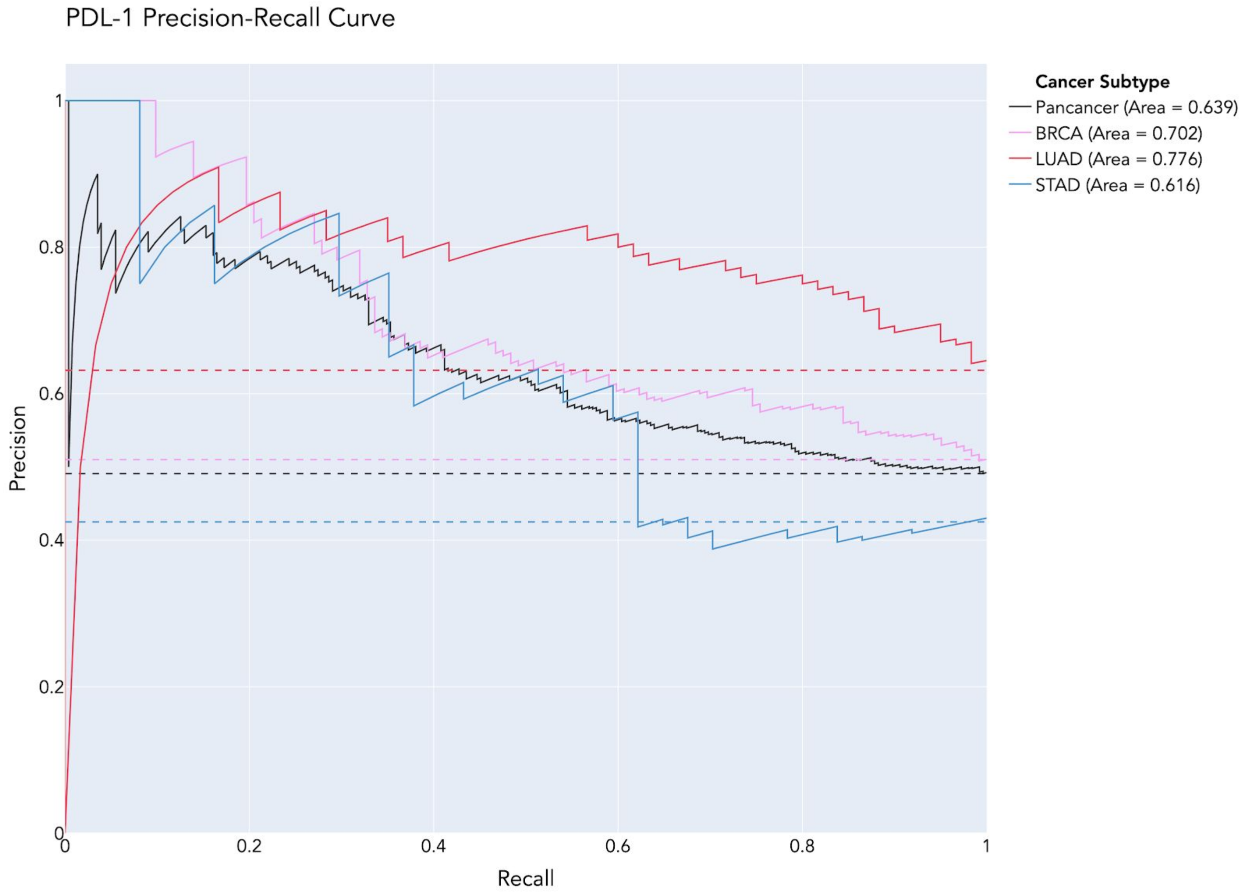
PDL-1 Precision-Recall Curve
Precision
Recall
0
0.2
0.4
0.6
0.8
1
Cancer Subtype
Pancancer (Area = 0.639)
BRCA (Area = 0.702)
LUAD (Area = 0.776)
STAD (Area = 0.616)

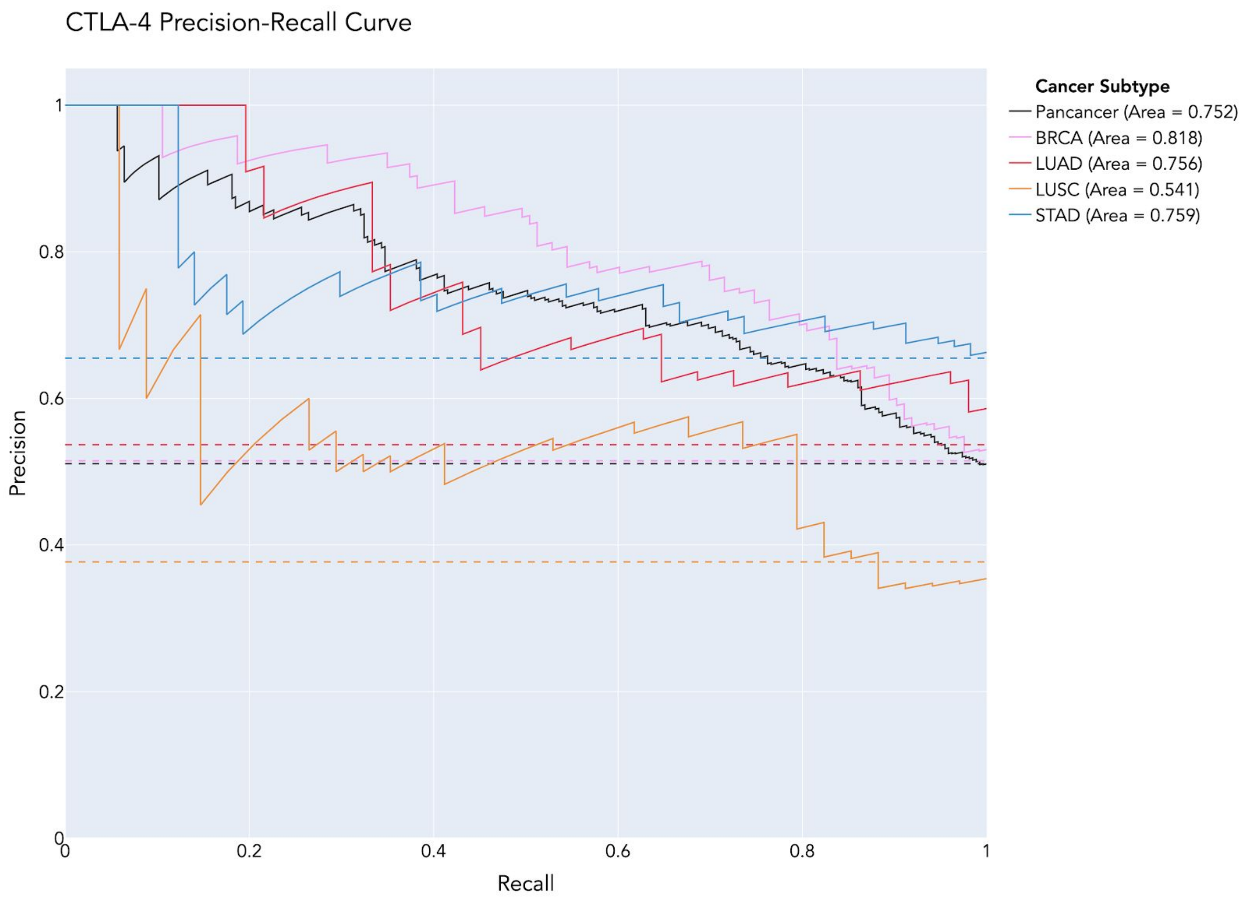

CTLA-4 Precision-Recall Curve
Cancer Subtype
Pancancer (Area = 0.752)
BRCA (Area = 0.818)
LUAD (Area = 0.756)
LUSC (Area = 0.541)
STAD (Area = 0.759)
Precision
Recall
0
0.2
0.4
0.6
0.8
1

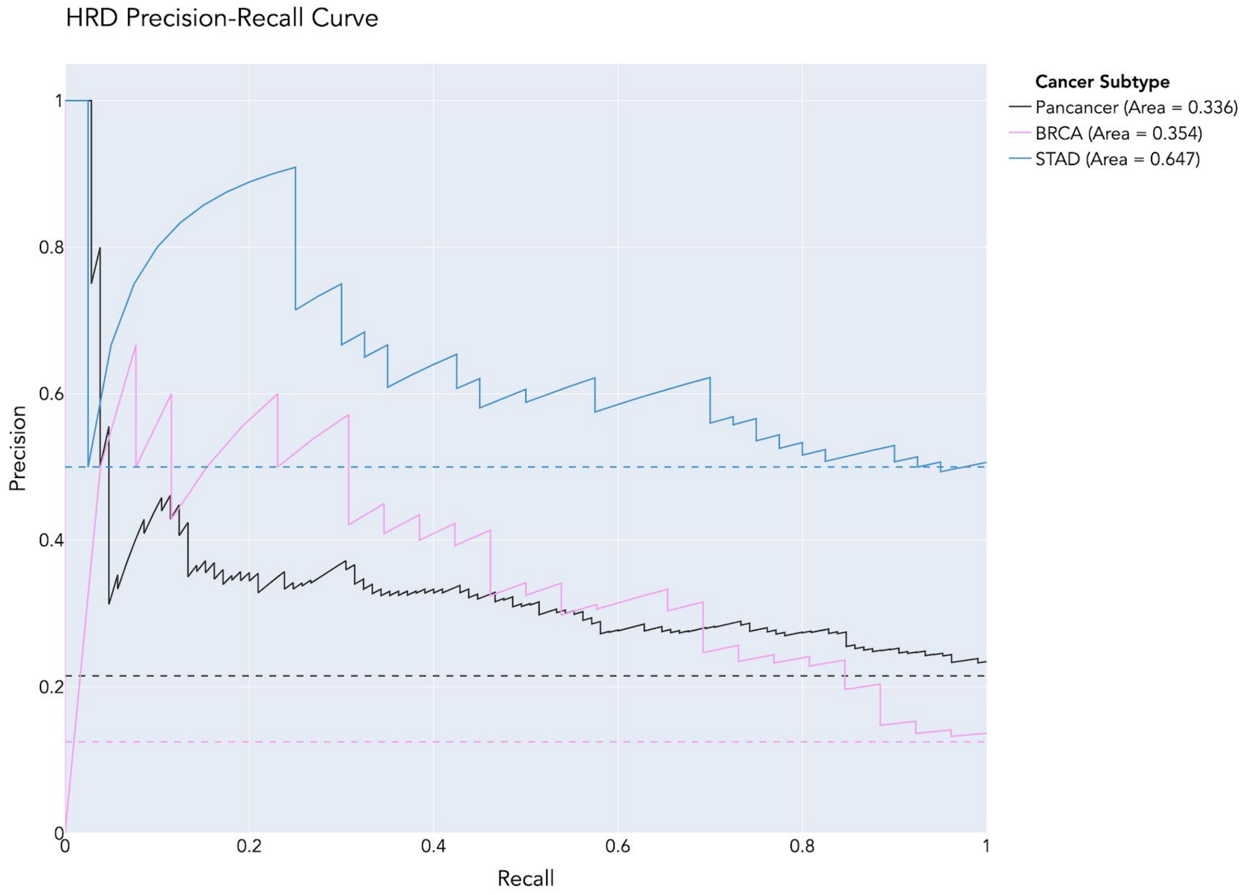
HRD Precision-Recall Curve
Cancer Subtype
Pancancer (Area = 0.336)
BRCA (Area = 0.354)
STAD (Area = 0.647)
Precision
Recall
0
0.2
0.4
0.6
0.8
1

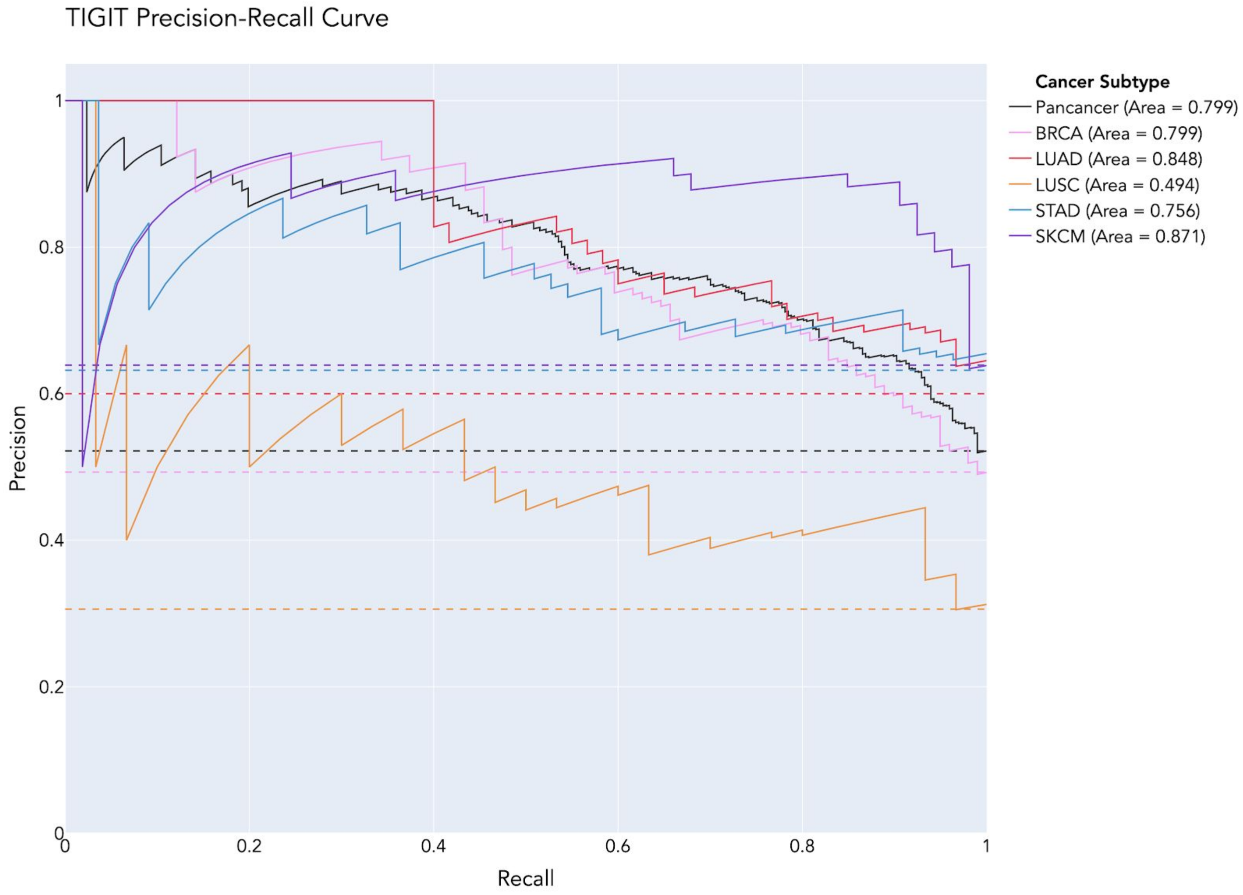

TIGIT Precision-Recall Curve
Precision
Recall
0
0.2
0.4
0.6
0.8
1
Cancer Subtype
Pancancer (Area = 0.799)
BRCA (Area = 0.799)
LUAD (Area = 0.848)
LUSC (Area = 0.494)
STAD (Area = 0.756)
SKCM (Area = 0.871)

Supplemental Figure 11. Predictive HIF clusters for cancer type-specific models
Boxplots of the top five most predictive HIF clusters (defined per cancer type) for predicting each molecular phenotype across cancer types and pan-cancer. Clusters are ranked by the maximum absolute beta across HIFs in a given cluster. Betas are computed per HIF as the average across the three models incorporated into the final ensemble evaluated on the hold-out set. The center and bounds of each boxplot represent the median and interquartile range (IQR; 25th, 75th percentiles) for HIF betas in each cluster, respectively. Upper and lower boxplot whiskers represent the smaller of the maximum beta value or the 75th percentile + 1.5 x IQR, and the larger of the minimum beta value or the 25th percentile - 1.5 x IQR, respectively. Each cluster is labeled with a representative HIF corresponding to the maximum absolute beta value. In cases in which that HIF is difficult to interpret, a more interpretable HIF within a five-fold difference of the maximum absolute beta is presented as indicated by a black asterisk. As the absolute value of beta values was used for ranking, HIFs with negative beta values are denoted by a red asterisk. For prediction tasks in which less than five HIF clusters have non-zero betas, only non-zero clusters are presented. Multiple predictive HIFs are visualized with overlaid cell or tissue-type heatmaps in Figure 3. Across all HIFs, tumor regions include cancer tissue (CT), cancer-associated stroma (CAS), and a combined CT+CAS. The sample size (number of HIFs) used to derive each boxplot is shown in the table below (for each boxplot graph, sample sizes are listed from top to bottom).

PD-1	Pancancer	n = 32, 49, 32, 9, 11
	BRCA	n = 10, 50, 31
	LUAD	n = 21, 22, 1, 25, 4
	LUSC	n = 2, 6, 5, 16, 7
	STAD	n = 2, 2, 6, 24, 6
PD-L1	Pancancer	n = 8, 30, 49, 20, 70
	BRCA	n = 24, 107, 7, 1, 69
	LUAD	n = 1, 30, 16, 15, 23
	STAD	n = 47, 5, 35, 12, 52
CTLA-4	Pancancer	n = 38, 4, 14, 77, 20
	BRCA	n = 95, 13, 13, 34, 53
	LUAD	n = 1, 22, 15, 4, 26

	LUSC	n = 1, 2, 1, 7, 1
	STAD	n = 30, 11, 15, 65, 30
HRD	Pancancer	n = 7, 15, 11, 8, 19
	BRCA	n = 20, 31, 2, 7, 7
	STAD	n = 2, 5, 9, 3, 3
TIGIT	Pancancer	n = 26, 22, 2
	BRCA	n = 105, 21, 83, 13, 16
	LUAD	n = 21, 5, 1, 15, 77
	LUSC	n = 3, 2, 18, 2, 3
	SKCM	n = 7, 3, 4, 4, 19
	STAD	n = 2, 10, 10, 5, 19

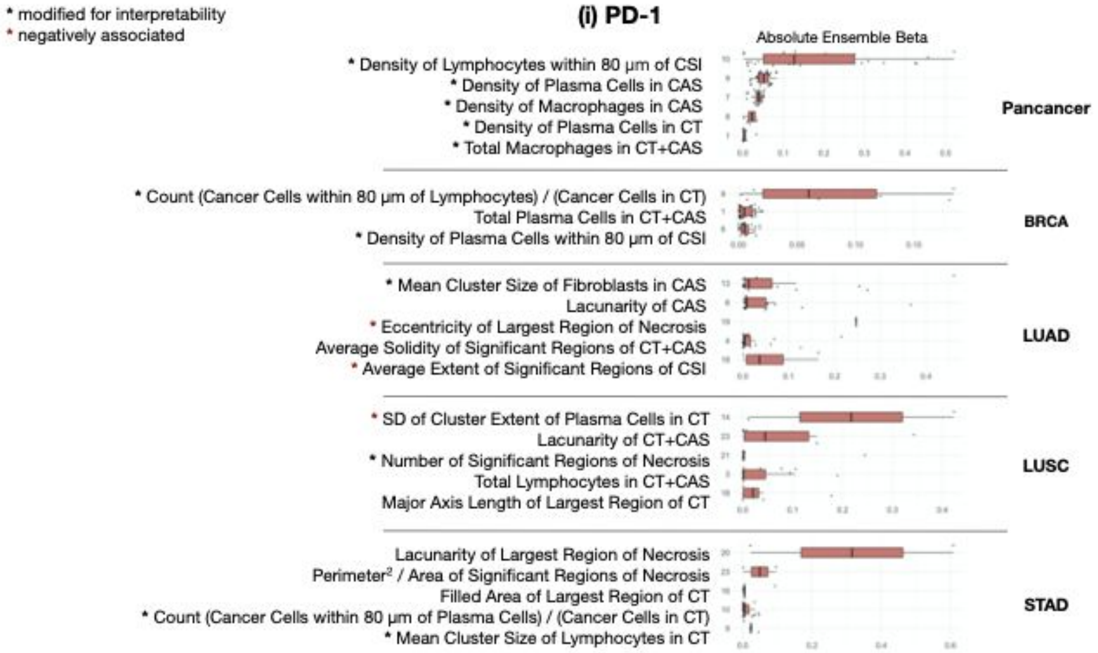

(ii) PD-L1

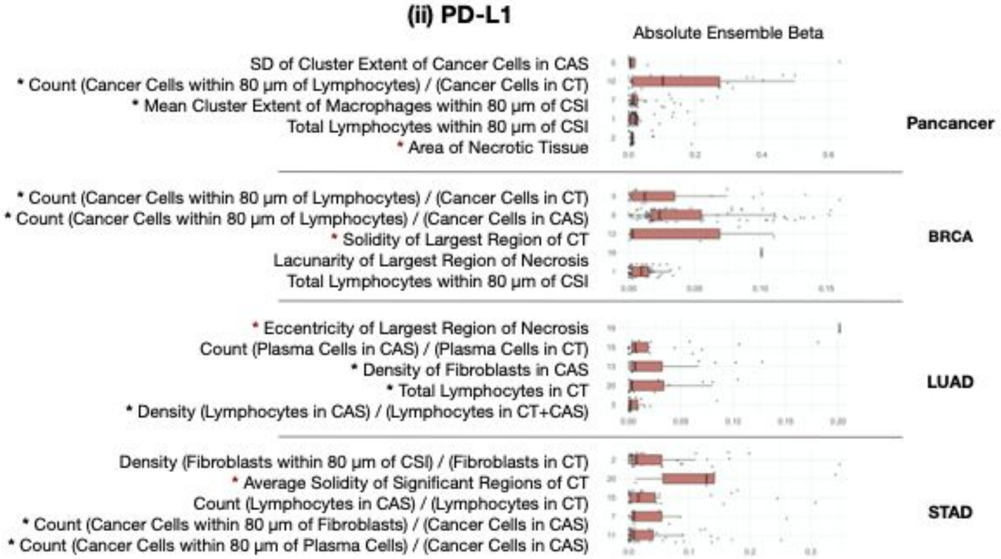

(iii) CTLA-4

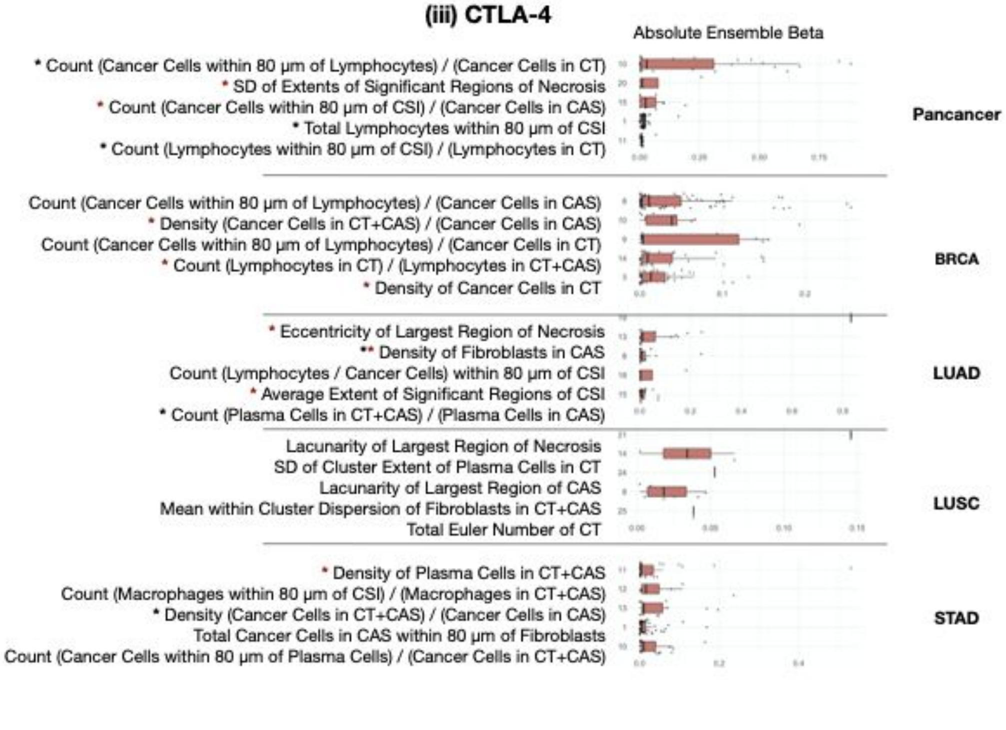

(iv) HRD

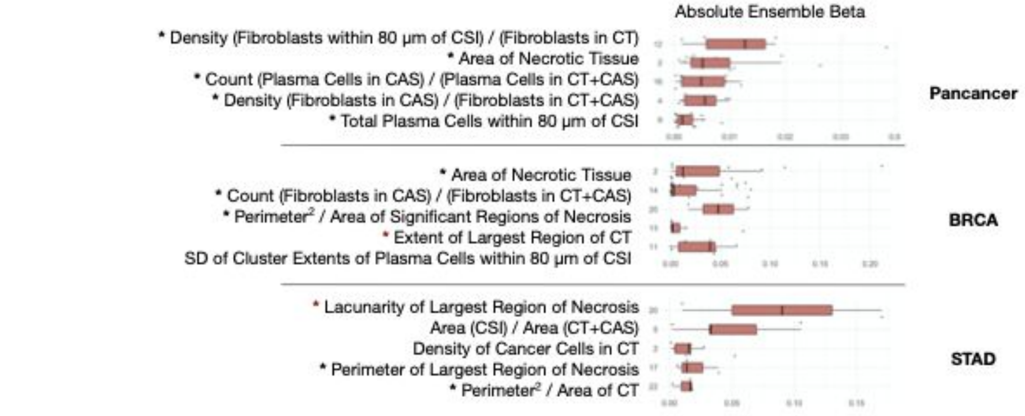

(v) TIGIT

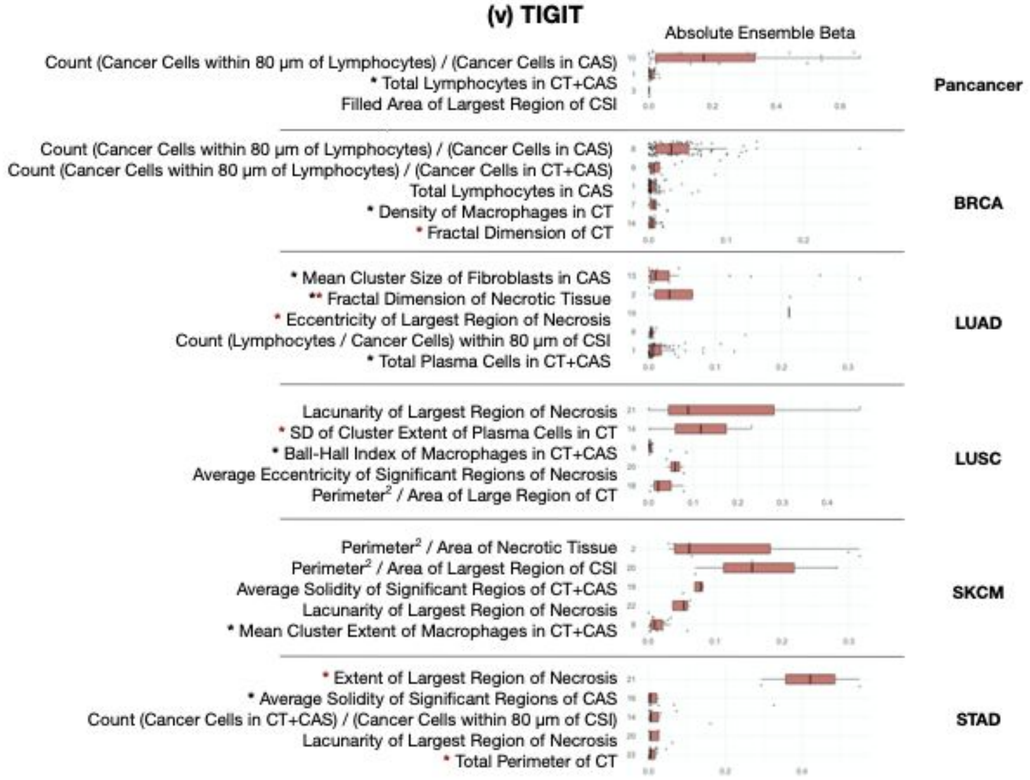

Supplemental Figure 12. Molecular phenotype concordance

Clustered heatmaps of concordance metrics between the five predicted molecular phenotypes computed on 1,893 patient samples across all five cancer types. In (a), Pearson correlations were computed between phenotypes in continuous form. In (b), the percentage agreement was computed between phenotypes after conversion into binary labels. We observe a strongly correlated cluster among the four immune checkpoint protein phenotypes (PD-1, PD-L1, CTLA-4, and TIGIT). HRD score appears relatively un-correlated with immune phenotypes.

a)

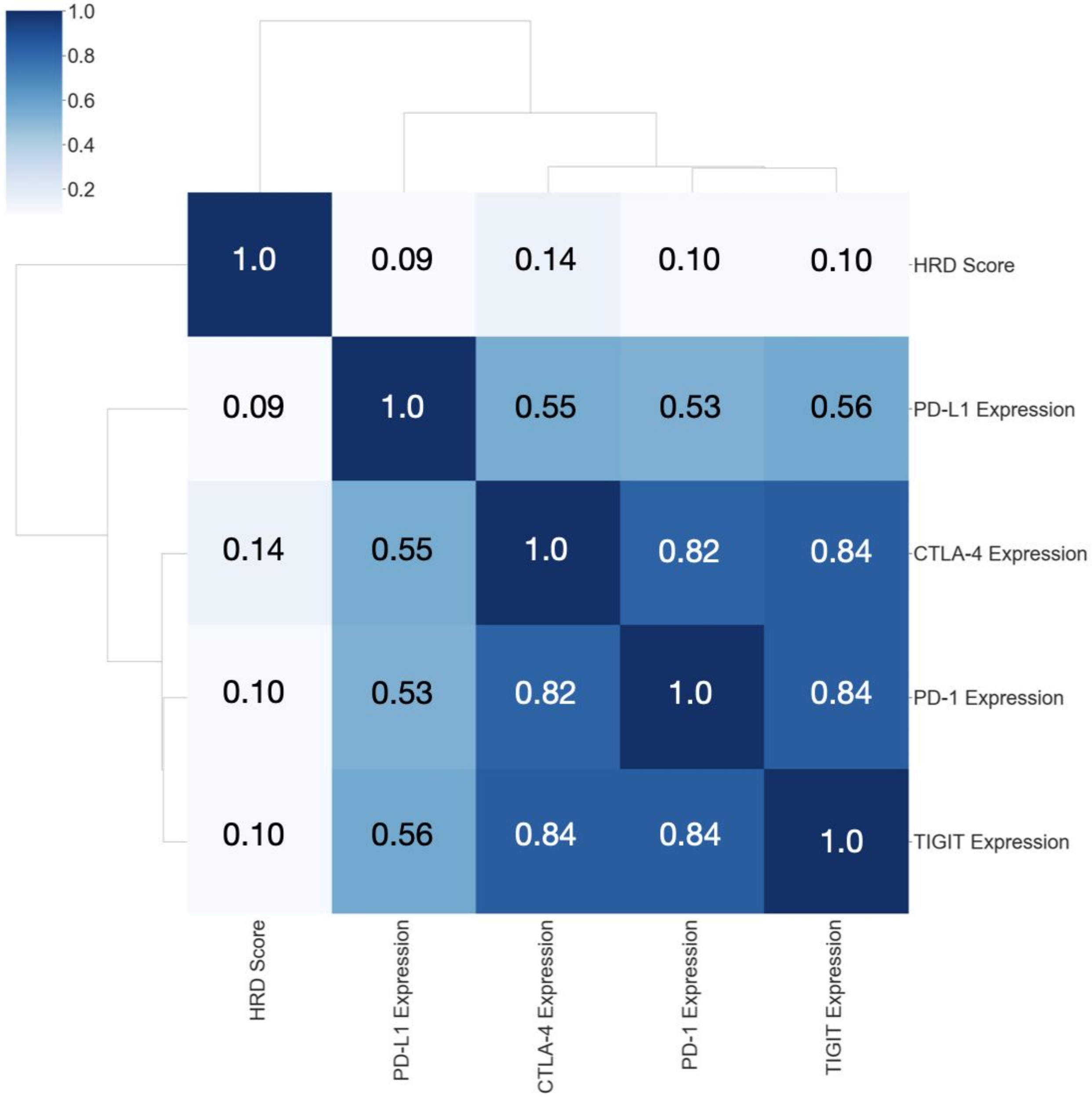

b)

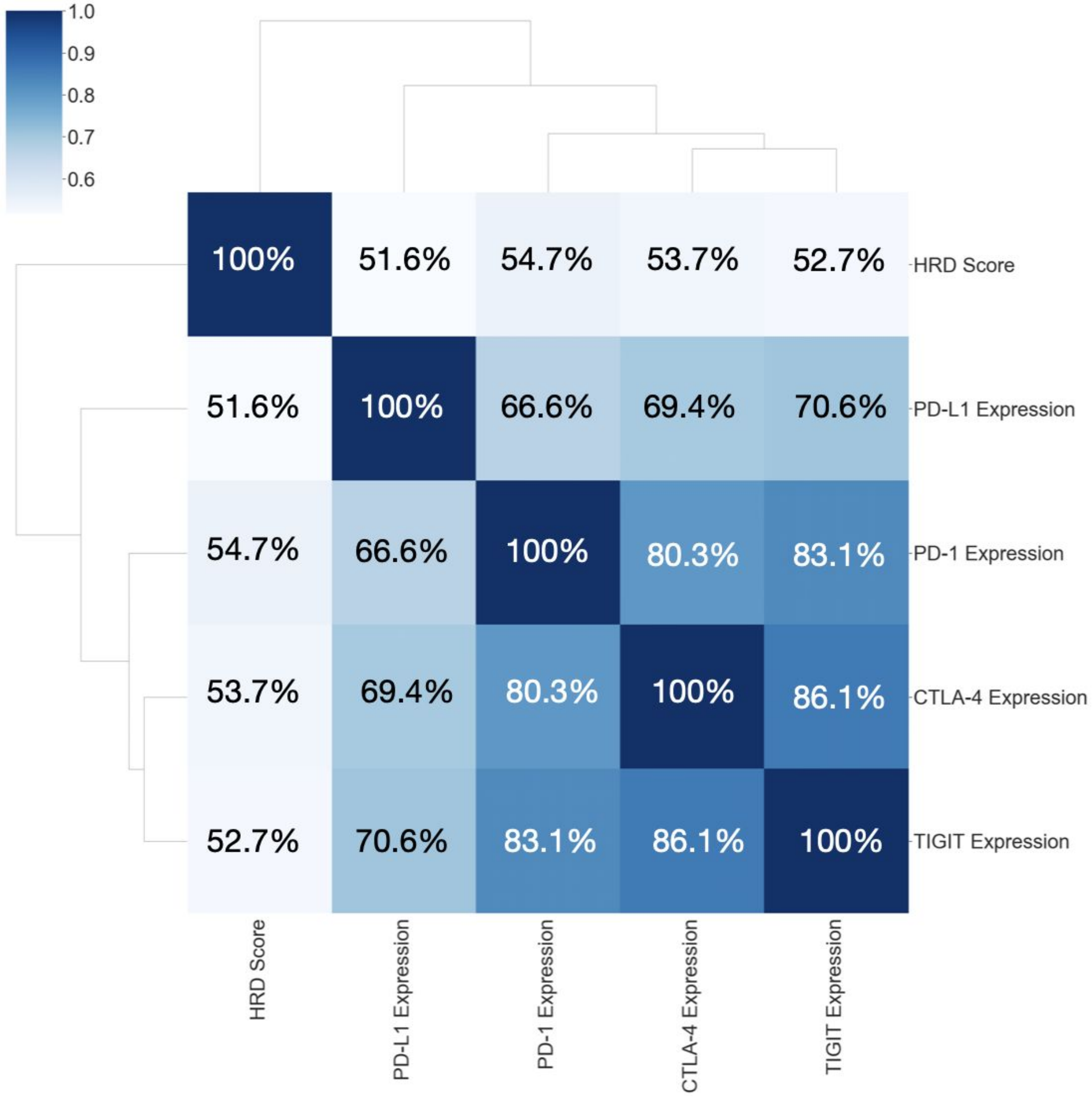
1.0
0.9
0.8
0.7
0.6
100%
51.6%
54.7%
53.7%
52.7%
51.6%
100%
66.6%
69.4%
70.6%
54.7%
66.6%
100%
80.3%
83.1%
53.7%
69.4%
80.3%
100%
86.1%
52.7%
70.6%
83.1%
86.1%
100%
HRD Score
PD-L1 Expression
PD-1 Expression
CTLA-4 Expression
TIGIT Expression
HRD Score
PD-L1 Expression
PD-1 Expression
CTLA-4 Expression
TIGIT Expression
